# Vaccination with *Mycoplasma pneumoniae* membrane lipoproteins induces IL-17A driven neutrophilia that mediates Vaccine-Enhanced Disease

**DOI:** 10.1038/s41541-022-00513-w

**Published:** 2022-07-29

**Authors:** Arlind B. Mara, Tyler D. Gavitt, Edan R. Tulman, Jeremy M. Miller, Wu He, Emily M. Reinhardt, R. Grace Ozyck, Meagan L. Goodridge, Lawrence K. Silbart, Steven M. Szczepanek, Steven J. Geary

**Affiliations:** 1 Department of Pathobiology and Veterinary Science, 61N Eagleville Rd. Unit-3089, Storrs, CT 06269 USA; 2 Center of Excellence for Vaccine Research (CEVR), 61N Eagleville Rd. Unit-3089, Storrs, CT 06269 USA; 3 Flow Cytometry Facility, Center for Open Research Resources and Equipment, 438 Whitney Rd Ext Unit 1149, Storrs, CT 06269 USA; 4 Connecticut Veterinary Medical Diagnostic Laboratory (CVMDL), 61N Eagleville Rd. Unit-3089, Storrs, CT 06269 USA; 5 Department of Allied Health, University of Connecticut, 358 Mansfield Rd, Unit-1101, Storrs, CT 06269 USA

**Keywords:** Protein vaccines, Bacterial host response, Bacterial pathogenesis, Bacterial infection

## Abstract

Bacterial lipoproteins are an often-underappreciated class of microbe-associated molecular patterns with potent immunomodulatory activity. We previously reported that vaccination of BALB/c mice with *Mycoplasma pneumoniae* (*Mp*) lipid-associated membrane proteins (LAMPs) resulted in lipoprotein-dependent vaccine enhanced disease after challenge with virulent *Mp*, though the immune responses underpinning this phenomenon remain poorly understood. Herein, we report that lipoprotein-induced VED in a mouse model is associated with elevated inflammatory cytokines TNF-α, IL-1β, IL-6, IL-17A, and KC in lung lavage fluid and with suppurative pneumonia marked by exuberant neutrophilia in the pulmonary parenchyma. Whole-lung-digest flow cytometry and RNAScope analysis identified multiple cellular sources for IL-17A, and the numbers of IL-17A producing cells were increased in LAMPs-vaccinated/*Mp*-challenged animals compared to controls. Specific IL-17A or neutrophil depletion reduced disease severity in our VED model—indicating that *Mp* lipoproteins induce VED in an IL-17A-dependent manner and through exuberant neutrophil recruitment. IL-17A neutralization reduced levels of TNF-α, IL-1β, IL-6, and KC, indicating that IL-17A preceded other inflammatory cytokines. Surprisingly, we found that IL-17A neutralization impaired bacterial clearance, while neutrophil depletion improved it—indicating that, while IL-17A appears to confer both maladaptive and protective responses, neutrophils play an entirely maladaptive role in VED. Given that lipoproteins are found in virtually all bacteria, the potential for lipoprotein-mediated maladaptive inflammatory responses should be taken into consideration when developing vaccines against bacterial pathogens.

## Introduction

*Mycoplasma pneumoniae*, the etiologic agent of primary atypical pneumonia, is an important human pathogen that causes significant morbidity worldwide. It is the leading cause of pneumonia in school-aged children and young adults, causing an estimated 2 million cases of community-acquired pneumonia and roughly 100,000 adult hospitalizations annually in the United States alone^1,2^. *Mp* is also known to cause tracheobronchitis and pharyngitis, and a myriad of extrapulmonary conditions, including potentially lethal conditions affecting the cardiovascular and central nervous systems^1^. *Mp* infection has been recently found to be associated with chronic obstructive pulmonary disease and asthma exacerbation, as evidenced by studies indicating that ≥ 50% of patients with chronic asthma are PCR and/or culture positive for *Mp*^3–5^. *Mp* is spread person-to-person by aerosolized droplets that result from persistent coughing, and respiratory manifestations such as indolent tracheobronchitis and pharyngitis are thought to be major contributors to this spread^6,7^. Given that transmission is facilitated by close contact, *Mp* outbreaks typically occur in crowded conditions such as day-care centers, hospitals, military barracks, and college dormitories, with the incidence of disease exceeding 70% among exposed populations^8–11^. Many cases of *Mp* go undiagnosed or misdiagnosed due to inadequate diagnostic tests, often resulting in mistreatment, and potentially contributing to the rise of antibiotic resistant strains^2,12–14^. Atypical pneumonia due to *Mp* infection has also been a major cause of morbidity in, and thus affects readiness of, military populations; having caused 61% of prolonged, non-productive coughs in US soldiers deployed in South Korea, 42% of hospitalizations in US soldiers with pneumonia during the Vietnam War, and 22% of respiratory infections among all US military personnel during Operation Enduring Freedom^15–17^. Naturally acquired immunity from previous *Mp* infections tends to be short lived, and recurrent *Mp* infections of an individual can occur^1,2^. Taken together, the burden of *Mp* on public health, along with limited preventative and therapeutic measures against *Mp*, have led to renewed interest in the development of an effective *Mp* vaccine, yet no vaccines are currently available in part due to *Mp* Vaccine-Enhanced Disease (VED).

*Mp* VED was first reported in human volunteers in the 1960s when federal prisoners were vaccinated with a formalin-inactivated *Mp* vaccine then challenged with a virulent *Mp* strain. While protective effects were observed in some participants, 36% of vaccinated individuals exhibited more severe clinical symptoms upon challenge than those receiving a placebo^18,19^. This VED phenomenon has created a major roadblock to additional *Mp* vaccine studies. Recent studies have recapitulated *Mp* VED in a BALB/c mouse model of respiratory *Mp* infection, utilizing live-attenuated vaccines or crude *Mp* protein extract^20–23^. In this VED model, vaccinated mice exhibit more severe lung histopathology post-challenge than unvaccinated mice, and lesion severity is associated with elevated IL-17A^20–24^. Using the BALB/c model, we recently reported that vaccination with *Mp* LAMPS caused VED that is dependent on the lipid moieties of *Mp* lipoproteins; however, the immunological mechanisms behind this phenomenon remain poorly understood^25^. Understanding these mechanisms remains of utmost importance as it can serve to inform the design of a safe and efficacious vaccine candidate.

Mycoplasma lipoproteins are known to drive immune responses upon binding to TLR-2 (in conjunction with TLR-1 for triacylated lipoproteins and TLR-6 for diacylated lipoproteins) expressed on many cell types, resulting in the production of inflammatory cytokines such as TNF-α and IL-6 among others^26^. *Mp* lipoproteins have also been shown to activate the NLRP3 inflammasome and induce the expression of IL-1β in a Gasdermin D and pyroptosis independent manner^27–29^. TLR-2 stimulation by lipoproteins has furthermore been linked to the expression of IL-23 by activated macrophages, which itself induces production of IL-17A by CD4 + T cells and subsequent IL-17A-driven neutrophil granulopoiesis, recruitment, and activation^30–32^. Neutrophils contribute to lung lesions, lung injury, and overall disease severity during pulmonary mycoplasmoses in multiple natural hosts, have been correlated with *Mp* VED in mouse models, and have been associated with more severe *Mp* disease in humans^20–24,33–47^. We therefore hypothesized that *Mp* vaccine candidates containing *Mp* lipoproteins activate IL-17A responses which trigger neutrophil recruitment (likely through the induction of the human IL-8 homolog, KC). Given that *Mp* is resistant to phagocytosis and killing by neutrophils, their activation likely contributes to the observed immunopathology in lipoprotein-induced *Mp* VED^48,49^.

## Results

### Inflammatory cytokines are elevated in lipoprotein-induced Mp VED

**Fig. 1:**
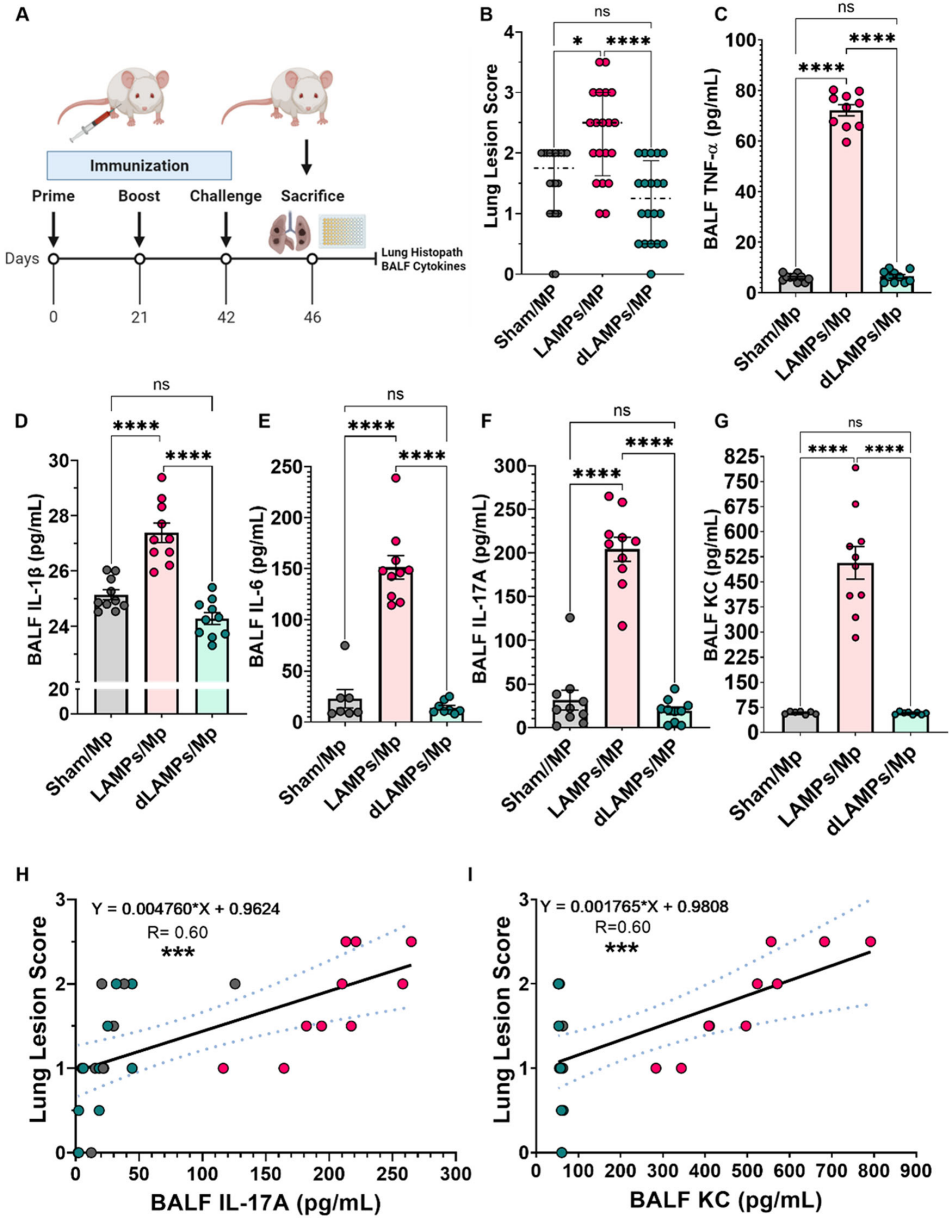
Vaccination with *M. pneumoniae* lipoproteins is associated with potent reactivation of inflammatory cytokines responses after challenge. **A** Illustration of experimental timeline and outcome measures. **B** Lung lesion scores of vaccinated-then-challenged animals. Bronchoalveolar lavage fluid (BALF) concentrations of **C** TNF-α, **D** IL-1β, **E** IL-6, **F** IL-17A, and **G** KC in vaccinated-then-challenged animals. Positive correlations between disease severity (lung lesion scores) and BALF **H** IL-17A, and **I** KC concentrations. *p < 0.5, **p < 0.1, ***p < 0.01, ****p < 0.001. Error bars for **B** indicate median and interquartile range and mean and SEM for **C**–**G**. Dotted lines for linear regression graphs indicate 95% confidence intervals. Each point represents data from an individual animal. Nonparametric lesion score data were analyzed via a one-way ANOVA on ranks (Kruskal–Wallis) with a Dunn’s post-hoc test for multiple pairwise comparisons. Parametric cytokine concentration data were analyzed via an ordinary one-way ANOVA with a Tukey’s post-hoc test for multiple pairwise comparisons. Linear regression was utilized to establish correlations.

To assess immune responses in our lipoprotein-induced *Mp* VED model, we vaccinated mice with saline as a placebo, lipid-associated membrane proteins (LAMPs, which contain lipoproteins and other transmembrane proteins) or with delipidated LAMPs (dLAMPs) produced via enzymatic de-acylation (Fig. 1A, Supplementary Fig. 1). Mice were intranasally challenged with 10^8^ CFU of *Mp* strain PI1428 (day 42 post-vaccination), then humanely sacrificed (day 46) to assess cytokine concentrations in bronchoalveolar lavage fluids (BALFs) and for histopathologic assessment of lungs. As expected, vaccination with *Mp* LAMPs resulted in VED upon challenge, whereas vaccination with dLAMPs or sham (saline) vaccination did not (Fig. 1B, Supplementary Fig. 2.). The inflammatory cytokines TNF-α, IL-1β, IL-6, IL-17A and the potent neutrophil chemoattractant KC were significantly elevated in BALFs of LAMPs-vaccinated/*Mp*-challenged animals when compared to sham or dLAMPs-vaccinated/*Mp*-challenged animals (Fig. 1C–G). Positive correlations were found between these cytokines and lung lesion severity (Supplementary Fig. 3A–C), with the strongest to IL-17A and KC (Fig. 1H, I). A strong positive relationship also was observed between IL-17A and KC (Supplementary Fig. 3D), which was expected, as IL-17A is known to induce the production of IL-8 family chemokines such as KC^43^.

### Increased numbers of Th17 cells, and other IL-17A producing cells, were found in mice exhibiting lipoprotein-induced Mp VED

**Fig. 2:**
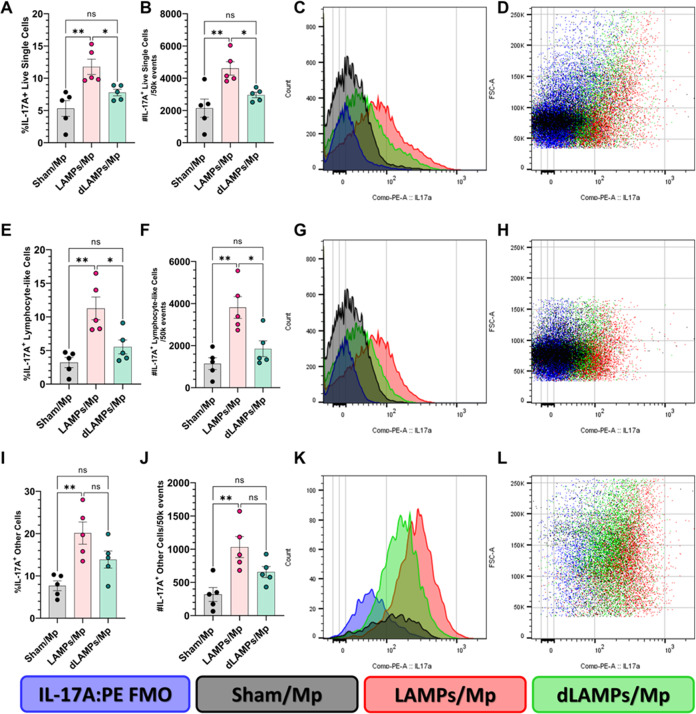
Numbers of IL-17A producing cells are elevated in LAMPs-vaccinated/Mp-challenged animals. Percentage (**A**, **E**, **I**) and number (**B**, **F**, **J**) of IL-17A positive cells when analyzing all live-single-cells (top), lymphocyte-like live-single cells (middle) and other-cells (bottom). Overlaid histograms (**C**, **G**, **K**) of cell count vs IL-17A signal on all live-single-cells (top), lymphocyte-like live-single cells (middle) and other-cells (bottom). Dot-plots (**D**, **H**, **L**) of Forward Scatter area vs IL-17A signal on all live-single-cells (top), lymphocyte-like live-single cells (middle) and other-cells (bottom). *p < 0.5, **p < 0.1, ***p < 0.01, ****p < 0.001. Error bars indicate mean and SEM. Each point represents data from an individual animal. Data from single representative animals from each vaccination group are shown on histograms and dot-plots. Nonparametric percent frequency data were analyzed via a one-way ANOVA on ranks (Kruskal–Wallis) with a Dunn’s post-hoc test for multiple pairwise comparisons. Parametric cell count data were analyzed via an ordinary one-way ANOVA with a Tukey’s post-hoc test for multiple pairwise comparisons.

**Fig. 3:**
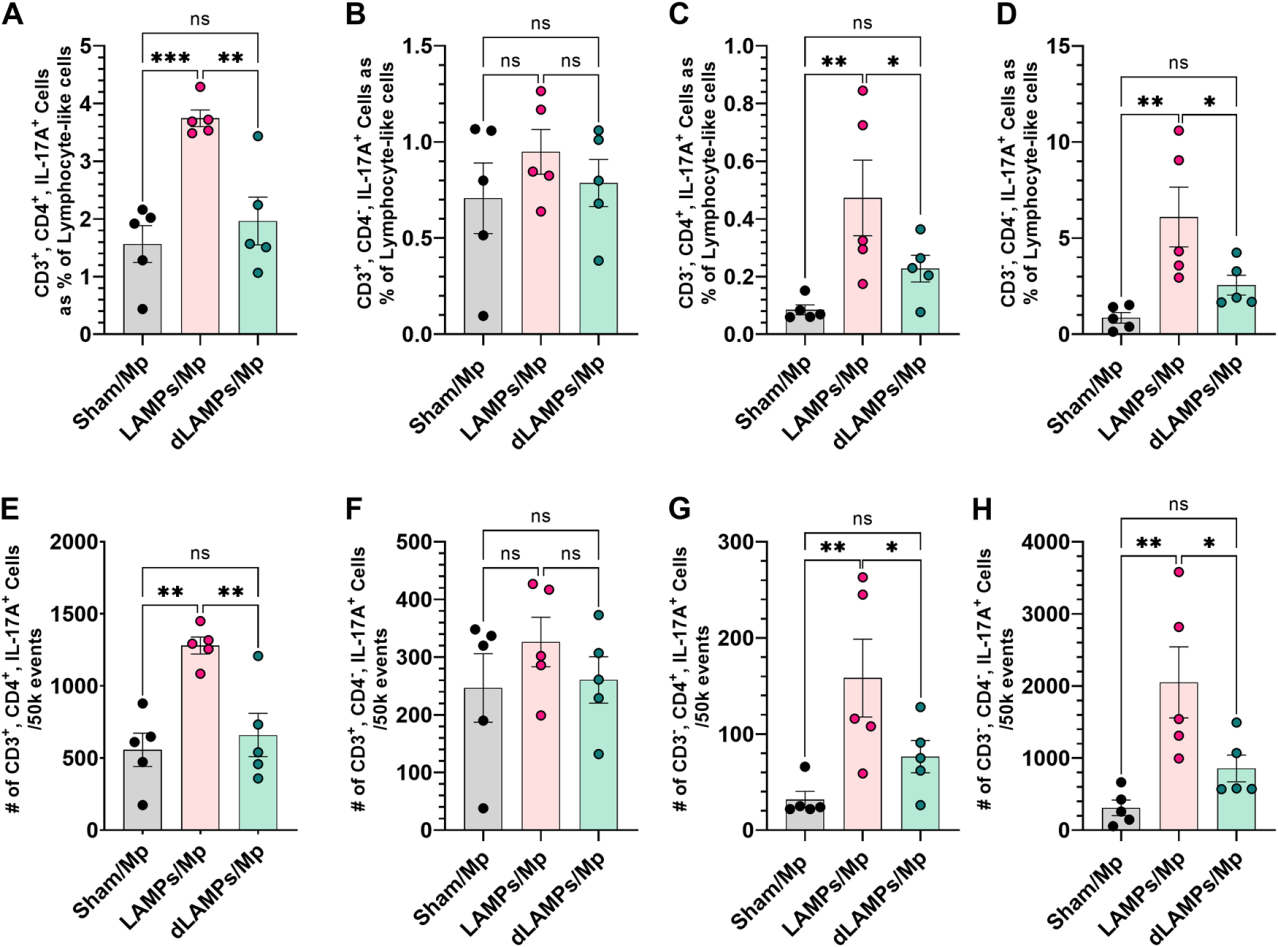
Numbers and percentages of IL-17A producing lymphocyte-like cells based on expression of CD3 and CD4. IL-17A producing CD3 + CD4 + cells as a percent of IL-17A positive lymphocyte-like cells (**A**) and raw counts per 50k analyzed events (**E**). IL-17A producing CD3 + CD4- cells as a percent of IL-17A positive lymphocyte-like cells (**B**) and raw counts per 50k analyzed events (**F**). IL-17A producing CD3-CD4 + cells as a percent of IL-17A positive lymphocyte-like cells (**C**) and raw counts per 50k analyzed events (**G**). IL-17A producing CD3-CD4- cells as a percent of IL-17A positive lymphocyte-like cells (**D**) and raw counts per 50k analyzed events (**E**). *p < 0.5, **p < 0.1, ***p < 0.01, ****p < 0.001. Error bars indicate mean and SEM. Each point represents data from an individual animal. Nonparametric percent frequency data were analyzed via a one-way ANOVA on ranks (Kruskal–Wallis) with a Dunn’s post-hoc test for multiple pairwise comparisons. Parametric cell count data were analyzed via an ordinary one-way ANOVA with a Tukey’s post-hoc test for multiple pairwise comparisons.

**Fig. 4:**
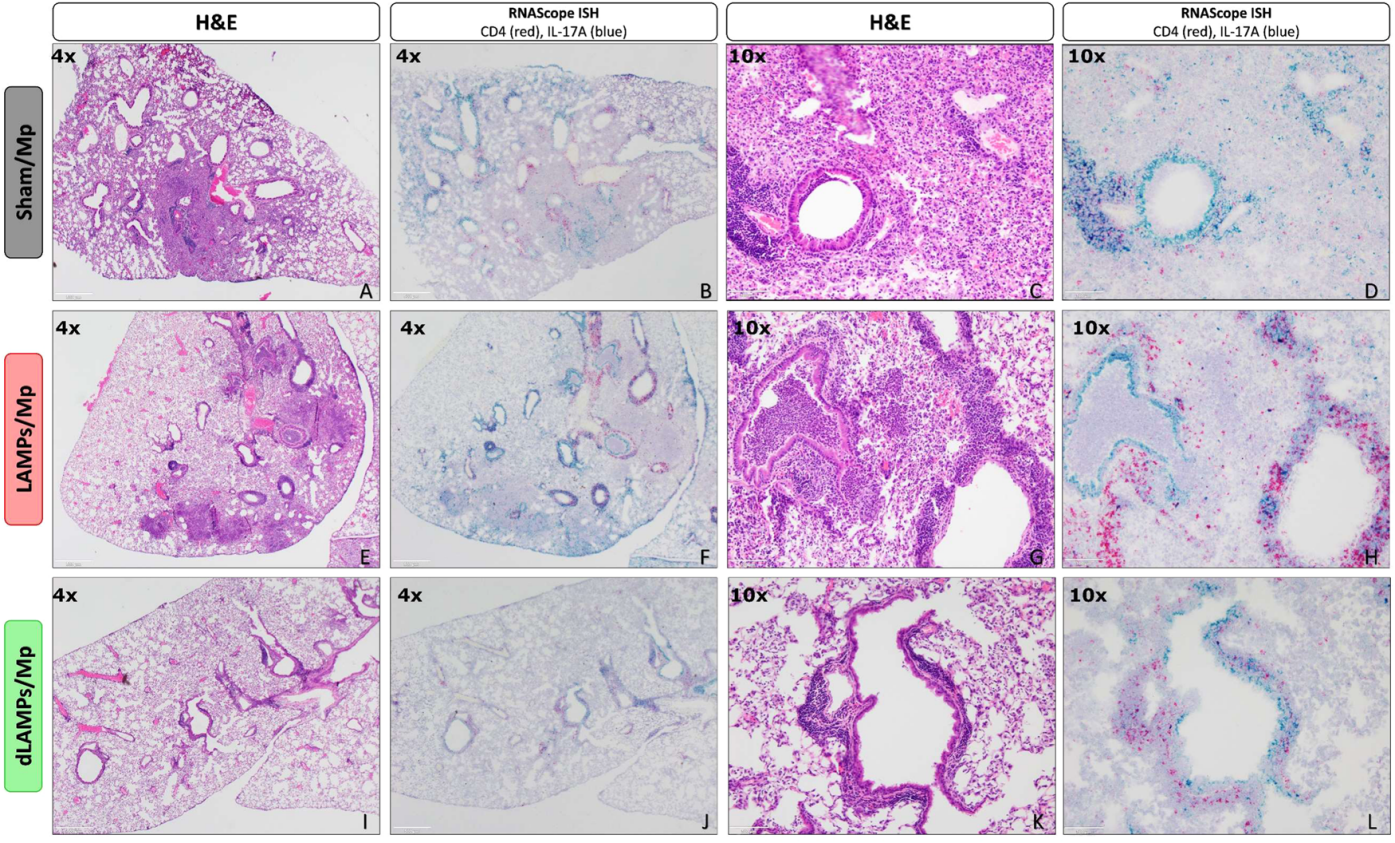
H&E and RNAScope in-situ hybridization lung histology from vaccinated-then-challenged animals. Representative H&E stained lung sections (**A**, **C**, **E**, **G**, **I**, **K**) and RNAScope in situ hybridization processed slides staining IL-17A transcript (blue) and CD4 transcript (red) (**B**, **D**, **F**, **H**, **J**, **L**) from Sham-vaccinated/Mp-challenged animals (top), LAMPs-vaccinated/Mp-challenged animals (middle) and dLAMPs-vaccinated/Mp-challenged animals (bottom). Scale bars indicate 500 um (4x) or 100 um (10x).

**Fig. 5:**
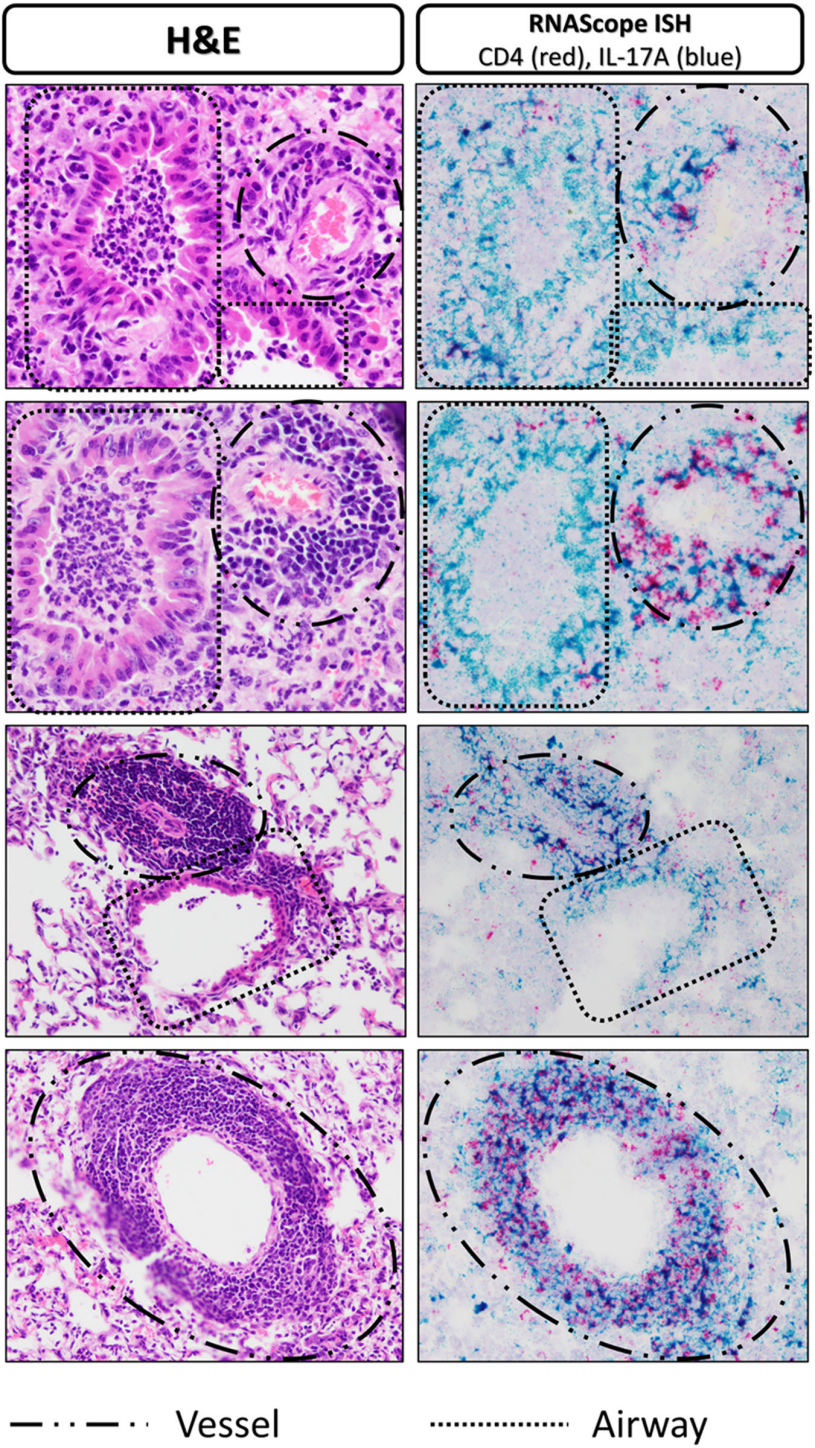
H&E and RNAScope in situ hybridization lung histology highlighting peribronchiolar and perivascular lesions. H&E stained lung sections (left) and RNAScope in situ hybridization processed slides staining IL-17A transcript (blue) and CD4 transcript (red) (right) displaying vessels and airways to show that CD4 mRNA and IL-17A mRNA co-localization was more frequent in the areas of perivascular cuffing.

Given the previous correlations between disease severity and IL-17A levels during Mycoplasma infection, and the elevated IL-17A in LAMPs-vaccinated/*Mp*-challenged animals, we wished to determine the cellular sources of IL-17A in our model. We performed flow cytometry on whole-lung-digests from Sham- LAMPs- and dLAMPs-vaccinated/Mp-challenged animals. After gating out debris, dead cells, and clumped cells we used size and granularity cut-offs to separate the live-single cells into a ‘lymphocyte-like’ population, and an ‘other-cells’ population (see gating strategy, Supplementary Fig. 4), then analyzed IL-17A expression in these populations. We found that percentages and numbers of IL-17A producing cells were elevated in LAMPs-vaccinated/*Mp*-challenged animals when compared to saline-vaccinated or dLAMPs-vaccinated -then-*Mp*-challenged animals. This was the case when considering all IL-17A^+ ‘^live-single-cells’ (Fig. 2A–D), as well as IL-17A^+^ ‘lymphocyte-like-cells’ and IL-17A^+^ ‘other-cells’ (Fig. 2E–L). When further subdividing the ‘lymphocyte-like’ population based on the expression of CD3 and CD4 (gating: Supplementary Fig. 5), we found that LAMPs-vaccinated/*Mp*-challenged animals had elevated numbers of IL-17A + CD3 + CD4 + cells, IL-17A + CD3-CD4 + cells, and CD3-CD4- cells when compared to Sham- or dLAMPs-vaccinated/*Mp*-challenged animals, but there were no significant differences in IL-17A + CD3 + CD4- cells among the groups (Fig. 3) CD3 + CD4 + cells appeared to be the primary contributors of IL-17A in Sham-vaccinated/*Mp*-challenged animals, accounting for roughly 51.42% (group average) of IL-17A + Lymphocyte-like cells while they accounted for 35.72% and 35.06% of IL-17A + Lymphocyte-like cells in LAMPs- and dLAMPs-vaccinated/*Mp*-challenged animals respectively, with CD3-CD4- populations making up the majority of the IL-17A + lymphocyte-like cells in the latter groups (Supplementary Fig. 6). Further investigation is needed to determine whether these cells represent a homogenous or heterogenous population of IL-17A producing cells, however. LAMPs-vaccinated/Mp-challenged animals had a higher group mean IL-17A:PE Median Fluorescence Intensity (MFI) value in IL-17A^+^ live-single-cells, although this difference was only statistically significant when compared against the Sham-vaccinated/*Mp*-challenged group (Supplementary Fig. 7). At the transcript level, IL-17A mRNA was found both co-localized with CD4 mRNA, as well as outside of CD4-producing cells, further corroborating the flow cytometry data that indicate multiple sources of IL-17A (Fig. 4). Co-localization of CD4 mRNA with IL-17A was most frequently observed in the perivascular lesions (Fig. 5), while IL-17A transcript was also detected in myeloid cells infiltrating the lung parenchyma, and surprisingly, in bronchial epithelial cells (Fig. 4).

### Lipoprotein-induced Mp VED is associated with elevated BALF neutrophilia

**Fig. 6:**
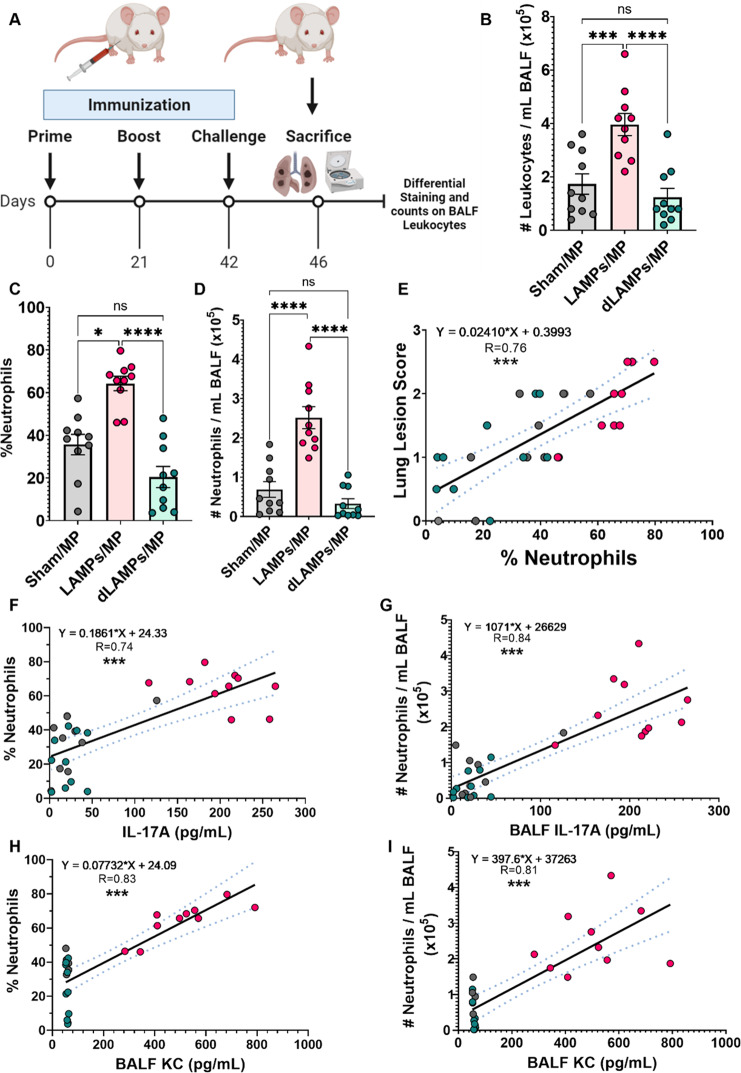
Vaccination with M. pneumoniae lipoproteins is associated with enhanced lung neutrophilia upon challenge, and this neutrophilia is associated with more severe disease. **A** Illustration of experimental timeline and outcome measures. **B** Numbers of lung-infiltrating leukocytes, proportion **C** and numbers **D** of lung-infiltrating neutrophils in vaccinated-then-challenged animals. **E** Positive correlations between lung-infiltrating neutrophil proportions and Lung Lesion Scores. **F**–**I** Correlations between proportions and numbers of lung-infiltrating neutrophils and IL-17A and KC concentrations. *p < 0.5, **p < 0.1, ***p < 0.01, ****p < 0.001. Error bars for **B**–**D** indicate mean and SEM. Dotted lines for linear regression graphs indicate 95% confidence intervals. Each point represents data from an individual animal. Nonparametric percent frequency/proportion data were analyzed via a one-way ANOVA on ranks (Kruskal–Wallis) with a Dunn’s post-hoc test for multiple pairwise comparisons. Parametric cell count data were analyzed via an ordinary one-way ANOVA with a Tukey’s post-hoc test for multiple pairwise comparisons. Linear regression was utilized to establish correlations.

To assess the composition of cells infiltrating the lung parenchyma during *Mp* VED, BALF differential cell counts were performed (Fig. 6A). BALF from LAMPs-vaccinated/*Mp*-challenged mice exhibited leukocytosis (Fig. 6B) primarily driven by an increase in neutrophils (Fig. 6A–D, Supplementary Fig. 8). No significant differences were observed in the numbers of mononuclear phagocytes (macrophages/monocytes), lymphocytes, or eosinophils (Supplementary Fig. 9). Furthermore, the observed neutrophilia was associated with higher lung lesions scores, indicating a positive relationship between infiltrating neutrophils and disease severity (Fig. 6E). Furthermore, strong positive correlations existed between proportions and numbers of BALF neutrophils and IL-17A and KC concentrations (Fig. 6F–I), indicating that neutrophils may be recruited through the actions of IL-17A and KC. Collectively, these data indicate that lipoprotein vaccination may induce *Mp* VED through IL-17A driven neutrophil recruitment.

### IL-17A neutralization reduces correlates of Mp VED, but impairs bacterial clearance

**Fig. 7:**
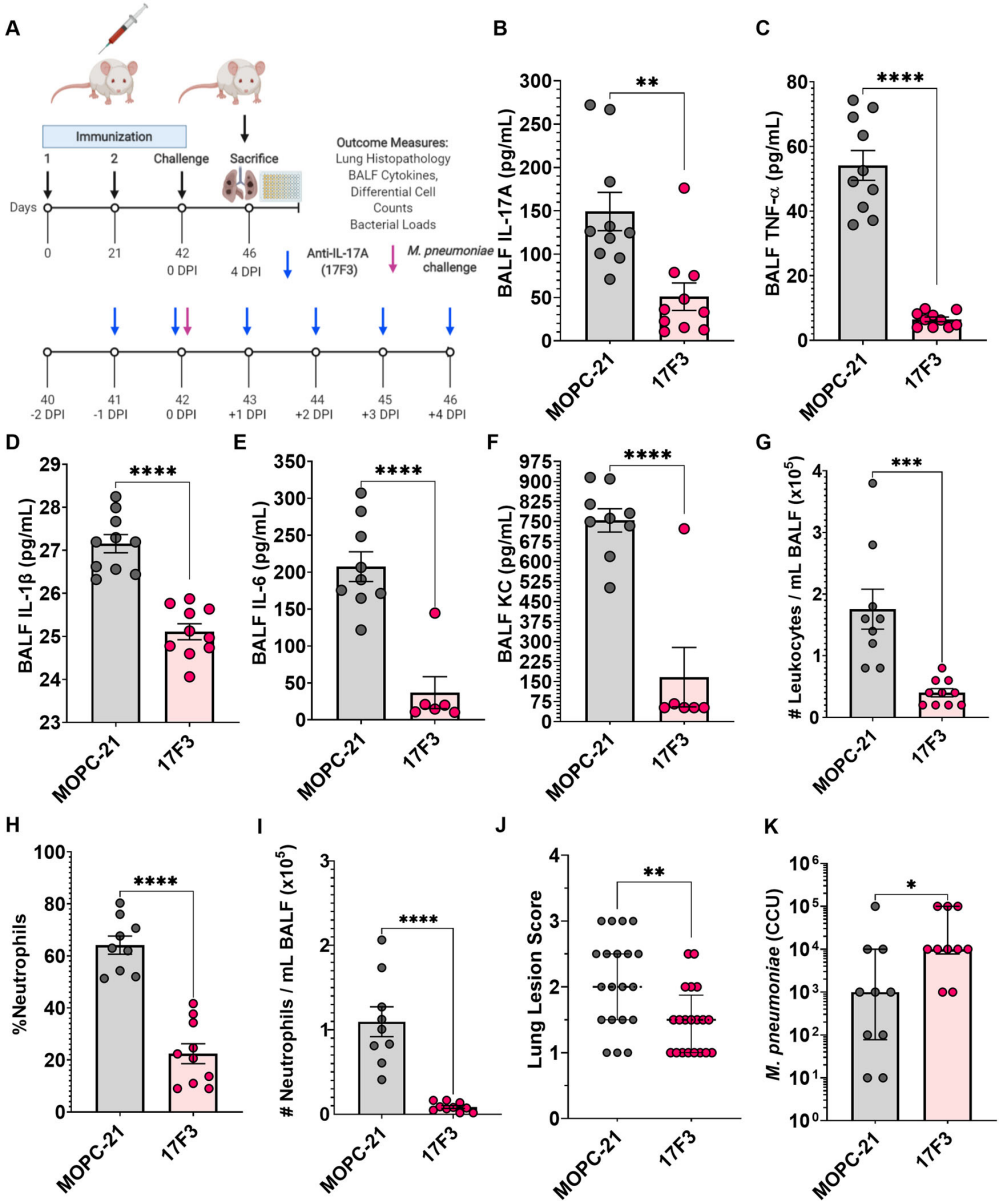
IL-17A neutralization in LAMPs-vaccinated/*Mp*-challenged animals reduces inflammatory cytokines, neutrophil recruitment, and severity of histopathological lung lesions but impairs bacterial clearance. **A** Illustration of experimental timeline and outcome measures. BALF concentrations of **B** IL-17A, **C** TNF-α, **D** IL-1β, **E** IL-6, and **F** KC in LAMPs-vaccinated/*Mp*-challenged animals receiving an anti-IL-17A neutralizing monoclonal antibody (17F3) or isotype control (MOPC-21). BALF numbers of lung-infiltrating leukocytes (**G**), and proportion of **H** and number of (**I**) lung-infiltrating neutrophils. **J** Lung Lesion Scores and **K** bacterial loads of vaccinated-then-challenged animals treated with anti-IL-17A antibody or isotype control. *p < 0.5, **p < 0.1, ***p < 0.01, ****p < 0.001. Error bars for **J** and **K** indicate median and interquartile range and mean and SEM for **B**–**I**. Each point represents data from an individual animal. Nonparametric lesion score, bacterial burden and percent frequency/proportion data were analyzed via an unpaired, two-tailed Mann–Whitney *U*-test. Parametric cytokine concentration and cell count data were analyzed via an unpaired, two-tailed *t*-test.

To determine whether IL-17A plays a causative role in the induction of *Mp* VED, we utilized an anti-IL-17A monoclonal antibody (17F3) to neutralize IL-17A in LAMPs-vaccinated/*Mp*-challenged mice (Fig. 7A). Treatment with the anti-IL-17A antibody successfully reduced the BALF IL-17A concentrations of LAMPs-vaccinated/*Mp*-challenged mice when compared to an isotype control antibody (MOPC-21) (Fig. 7B). IL-17A neutralization reduced the levels of inflammatory cytokines TNF-α, IL-1β, IL-6, and the chemotactic factor KC (Fig. 7C–F), indicating that IL-17A precedes subsequent inflammatory events during *Mp* VED. Moreover, IL-17A neutralization resulted in a reduction of BALF leukocytosis (Fig. 7F) which was primarily driven by a decrease in neutrophils (Fig. 7H, I). A modest reduction in mononuclear phagocytes and lymphocytes was also observed (Supplementary Fig. 10). IL-17A neutralization also resulted in the reduction of lung pathology (Fig. 7J). Surprisingly, IL-17A neutralization impaired bacterial clearance, as there was a significant increase in *Mp* recovery from LAMPs-vaccinated/IL-17A neutralized/*Mp*-challenged mice when compared to LAMPs-vaccinated/isotype-treated/*Mp*-challenged animals (Fig. 7K, Supplementary Fig. 11).

### Neutrophil depletion ameliorates Mp VED immunopathology and bacterial burden

**Fig. 8:**
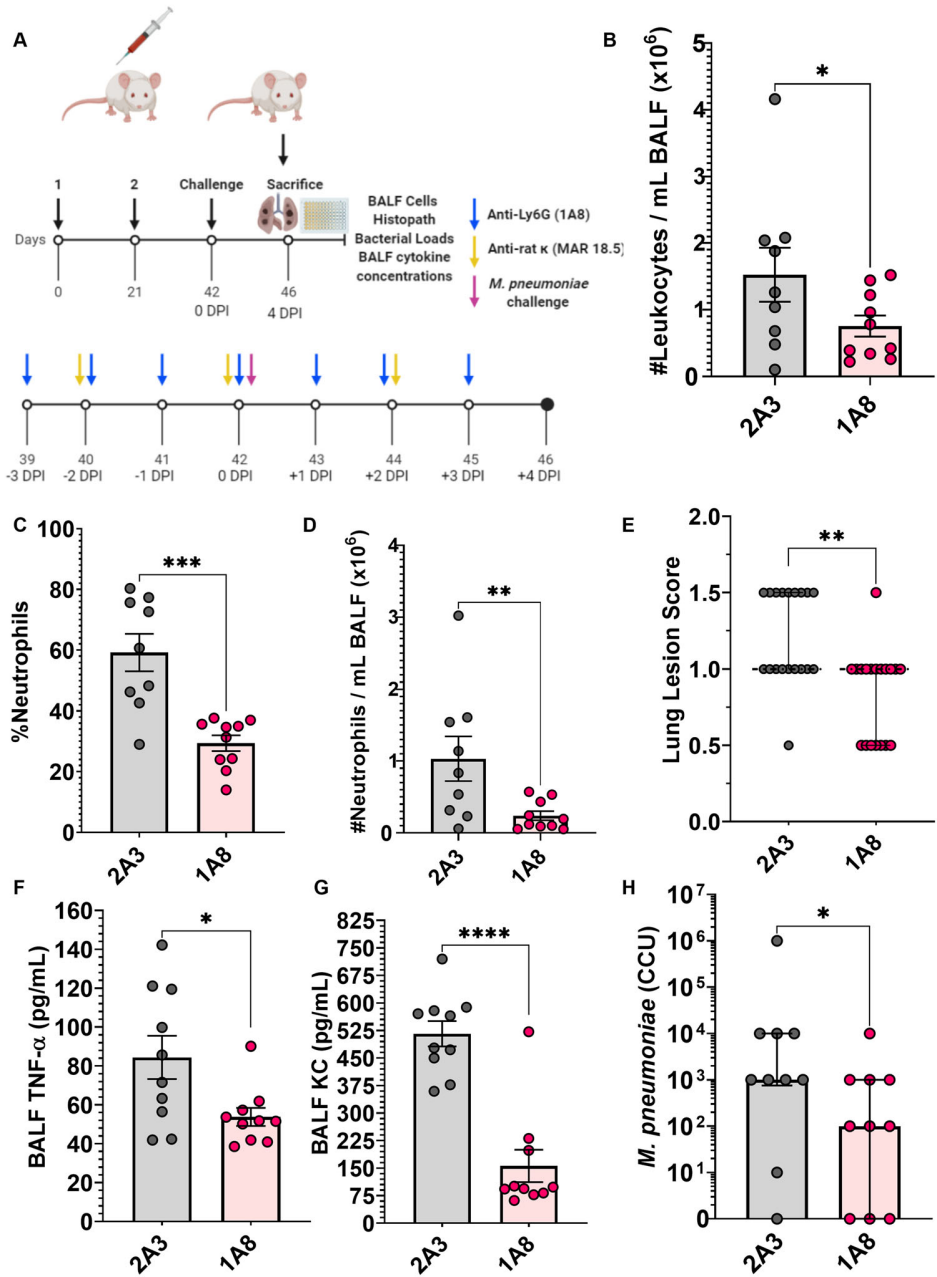
Neutrophil depletion in LAMPs-vaccinated/*Mp*-challenged mice ameliorates disease severity and enhances bacterial clearance. **A** Illustration of experimental timeline and outcome measures. Anti-Ly6G (1A8) antibody is given as a daily injection and its isotype control clone 2A3 is given to control animals following the same schedule. To induce an isotype switch of the 1A8 antibody for sustained neutrophil depletion, the anti-rat K (MAR18.5) antibody was given every-other day, while the isotype control MOPC-21 was given at the same schedule to control animals. To achieve sustained depletion of neutrophils BALF numbers of lung-infiltrating leukocytes (**B**), and proportion of **C** and number of **D** lung-infiltrating neutrophils in LAMPs-vaccinated/*Mp*-challenged animals receiving either neutrophil depletion antibodies (1A8-anti-Ly6G) or isotype control (2A3). BALF concentrations of **F** TNF-α and **G** KC in LAMPs-vaccinated/*Mp*-challenged animals receiving neutrophil depleting antibodies or isotype control. **E** Lung Lesion Scores and **H** bacterial loads of vaccinated-then-challenged animals treated with neutrophil depleting antibody or isotype control. *p < 0.5, **p < 0.1, ***p < 0.01, ****p < 0.001. Error bars for **E** and **H** indicate median and interquartile range and mean and SEM for **B**–**D** and **F**, **G**. Each point represents data from an individual animal. Nonparametric lesion score, bacterial burden and percent frequency/proportion data were analyzed via an unpaired, two-tailed Mann–Whitney *U*-test. Parametric cytokine concentration and cell count data were analyzed via an unpaired, two-tailed *t*-test.

To determine whether neutrophils play a causative role in *Mp* VED, monoclonal antibodies (anti-Ly6G antibody clone 1A8) were used to deplete neutrophils in LAMPs-vaccinated/*Mp*-challenged mice according to a previously reported strategy^50^, illustrated in Fig. 8A. Treatment with antibody 1A8 successfully reduced the number of lung-infiltrating leukocytes (Fig. 8B), and this reduction was entirely driven by the specific depletion of neutrophils (Fig. 8C, D) as the number of mononuclear phagocytes, lymphocytes, and eosinophils was unaffected (Supplementary Fig. 12). Neutrophil depletion also reduced lung lesion severity and concentrations of BALF TNF-α and KC; however, concentrations of IL-17A, IL-1β, and IL-6 were unaffected (Fig. 8E–G; Supplementary Fig. 13). Neutrophil depletion also improved bacterial clearance (Fig. 8H; Supplementary Fig. 14).

## Discussion

Bacterial lipoproteins are a class of microbe-associated molecular patterns (MAMPs) that induce potent immune responses following their recognition by TLR-2-containing complexes; with TLR-2/1 complexes recognizing triacylated bacterial lipoproteins, and TLR-2/6 complexes recognizing diacylated lipoproteins^51^. We have previously reported that vaccination with *Mycoplasma pneumoniae* lipoproteins resulted in vaccine-enhanced disease upon challenge, but the immune responses underpinning this phenomenon remained poorly understood^25^. Here we report that vaccination of BALB/c mice with *Mp* LAMPs results in exacerbation of lung pathology and the activation of inflammatory cytokines TNF-α, IL-6, IL-1β, and IL-17A and of the neutrophil chemoattractant KC upon challenge with *Mp*. These effects were clearly lipid moiety-dependent, as the responses in mice vaccinated with dLAMPs were comparable to those of controls. Positive correlations were observed between these cytokines and disease severity, with the strongest correlations being those between IL-17A and lesion severity, and between KC and lesion severity, indicating that these cytokines play a causative role in the induction of lung pathology in *Mp* VED.

Flow cytometry and RNAScope analysis of the lungs of vaccinated-then-challenged animals revealed multiple cellular sources of IL-17A, including lymphocyte-like populations and myeloid-like populations, with the numbers and percentages of IL-17A positive cells being significantly higher in mice exhibiting VED (LAMPs-vaccinated/*Mp*-challenged animals). Producers of IL-17A included populations such as CD3 + CD4 + lymphocyte-like cells which represent Th17 cells, CD3 + CD4− lymphocyte-like cells which could represent Tc17, γδ T cells or some NKT/iNKT cell populations, CD3-CD4 + lymphocyte-like cells which could represent ILC3s and some NK cell populations, CD3-CD4- lymphocyte-like cells which could be represented by B cells and CD4- NK cells^52–61^. Surprisingly, IL-17A transcript was also detected in bronchial epithelial cells, which are seldom reported to produce IL-17A but can do so when exposed to cigarette smoke or in a mouse model of COPD induced by using cigarette smoke and elastin^62^. In this context, epithelial IL-17A induced autocrine mucin (Muc5AC) expression and inflammatory cytokines IL-6, TNF-α, and IL-1β, which are also produced during *Mp* infection^63,64^. It is unclear how *Mp* infection can induce IL-17A expression in bronchial epithelial cells, although this expression is clearly dependent on infection of mice with *Mp*, rather than vaccination, as all *Mp*-challenged animals, regardless of vaccination status, had detectable IL-17A transcript in bronchial epithelium. While lipoprotein-vaccinated/*Mp*-challenged animals had a higher mean IL-17A MFI value than the sham control, this difference was not statistically different from dLA*MP*s-vaccinated/Mp-challenged animals, indicating that the differences in IL-17A concentrations among the treatment groups were likely due to the increase in the numbers of IL-17A-producing cells, rather than an increase in IL-17A production by any individual cell population. As the most robust IL-17A staining occurred in regions of perivascular cuffing, lesions which are more frequent in lipoprotein-vaccinated/*Mp*-challenged animals, it is possible that the increase in the numbers of these cells accounts for the excess IL-17A levels observed in lipoprotein-vaccinated/*Mp*-challenged animals.

IL-17A-driven KC production is known to contribute to neutrophil recruitment, and significant numbers of neutrophils were observed in the lung parenchyma of lipoprotein-vaccinated/*Mp*-challenged animals, further implicating IL-17A and neutrophils in the induction of *Mp* VED. We then showed that IL-17A and neutrophils play a causative role in exacerbating lung pathology during *Mp* VED, as either IL-17A neutralization or neutrophil depletion in lipoprotein-vaccinated/*Mp*-challenged animals each abrogated exacerbation of lung pathology. Importantly, IL-17A neutralization resulted in a reduction in BALF cytokines TNF-α, IL-1β, IL-6, and KC and concomitant reduction in neutrophil recruitment, which indicates that IL-17A contributes to the immunopathology observed in *Mp* VED by inducing neutrophil recruitment through KC. Surprisingly, IL-17A neutralization also impaired bacterial clearance, which may be due to the modestly reduced numbers of phagocytic cells, such as monocytes, observed during IL-17A neutralization. As IL-17A has also been shown to induce the expression of mucin and antimicrobial peptides by airway epithelium, it may also be likely that the increase in bacterial burden may be due to the loss of these important mediators of barrier immunity^65,66^. Depletion of neutrophils also abrogated exacerbation of lung pathology, but also surprisingly improved bacterial clearance, indicating that neutrophils play an entirely maladaptive role in *Mp* VED, serving to both exacerbate immunopathology and impair bacterial clearance. This contrasts IL-17A, which exacerbates immunopathology but contributes to bacterial clearance—thus conferring both maladaptive and protective roles in *Mp* VED. A previous study indicated that neutrophils simply did not significantly contribute to *Mp* clearance in *Mp*-challenged naïve mice, though they did not find bacterial clearance to be improved when neutrophils were depleted^48^. This study was conducted in C57BL/6 mice however, which tend to be more resistant to infection with *Mp*, while our study was conducted in BALB/c mice which tend to be more susceptible. It is possible therefore that mouse strain-differences in neutrophil populations may explain why we see an inhibition in bacterial clearance by neutrophils in our model. An alternative, and more likely explanation, is also that the exuberant recruitment of neutrophils in mice experiencing *Mp* VED sterically prohibits clearance of *Mp* by other protective cells, such as macrophages. *Mp* are resistant to killing by neutrophils, in part due to their ability to degrade neutrophil extracellular traps using Mpn491, a secreted nuclease^67^. It is likely, therefore, that when the predominant cells in the lung parenchyma are neutrophils that do not contribute to clearance (which is the case in mice with *Mp* VED), *Mp* are able to better colonize the host and replicate. This could explain why we see improved bacterial clearance when we deplete neutrophils in our model.

**Fig. 9:**
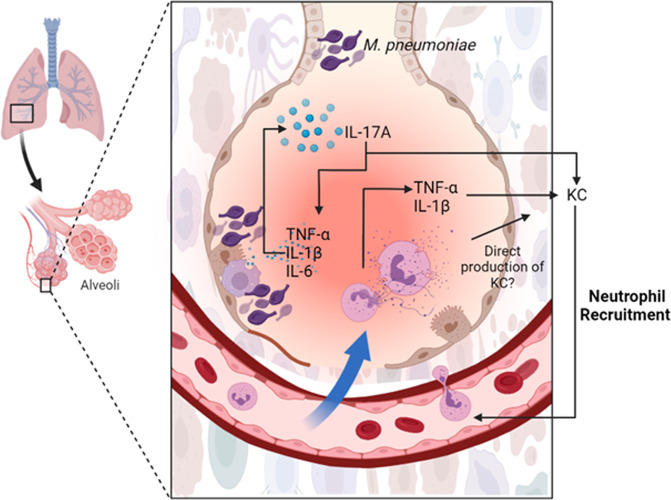
Putative model of *M. pneumoniae* Vaccine-Enhanced Disease. Anamnestic reactivation of IL-17A recall responses in LAMPs-vaccinated/*Mp*-challenged animals results in the further production of TNF-α, IL-1β, IL-6, and KC. TNF-α and IL-1β can further induce the expression of the neutrophil chemotactic factor KC (Supplementary References 1,2), and in the presence of IL-6, further potentiate IL-17A production by helper T-cells (Supplementary Reference 3), establishing a positive feedback loop of neutrophil recruitment and inflammation. Neutrophils also contribute to TNF-α production which can further potentiate KC production, contributing to the positive neutrophil recruitment loop that is associated with the more severe disease observed in *Mp* VED. (*Created in biorender.com by ABM*).

Based on these observations, and previously published studies, we propose the following model for *Mp* VED (Fig. 9). Vaccination with lipoprotein-containing *Mp* vaccines results in anamnestic responses characterized by overexuberant expression of TNF-α, IL-1β, IL-6, and, importantly, IL-17A and KC. These then result in lung neutrophilia and lung injury after activation by *Mp* lipoproteins^49^. Given that *Mp* is resistant to neutrophil-mediated killing^48^, the activation of neutrophils results in immunopathology rather than bacterial clearance. Our data also indicate that neutrophils contribute to the production of TNF-α which, along with IL-1B and IL-17A, can induce KC production, potentially resulting in a positive neutrophil recruitment loop that causes immunopathology^68,69^. TNF-α, IL-1β, and IL-6 can also further promote Th17 cell differentiation, potentially contributing to the IL-17A/neutrophil recruitment loop that mediates disease severity^70^. In addition to inducing KC production, IL-17A can also contribute to the exuberant neutrophilia via emergency neutrophil granulopoiesis through the induction of G-CSF^71^, but further experimentation is required to show if this is the case during *Mp* VED as well.

Our data provide a potential explanation as to why *Mp* VED occurred in human volunteers receiving a formalin-inactivated vaccine^18,19^. Innate cytokines such as TNF-α and IL-1β, which are elevated in mice experiencing *Mp* VED, are also associated with more severe *Mp* disease in human patients, especially in children^36,72–80^. Furthermore, the expression of type 17 cytokines such as IL-23, IL-17, and the IL-17A-induced neutrophil chemotactic factor IL-8 are also elevated in patients with severe *Mp* infections^36–47,72,81^, indicating that elevated Th17 responses may contribute to disease severity. It appears, therefore, that IL-17A constitutes a double-edged sword in host response to *Mp;* exacerbating immunopathology through neutrophil recruitment, but also controlling bacterial burdens through other (currently unknown) mechanisms.

In the context of *Mp*, neutrophils are maladaptive, contributing to the enhancement of lung lesions through their recruitment to the lung, yet not contributing to bacterial clearance. Indeed, neutrophils have been found to induce lung damage in mice challenged with *M. pneumoniae*^82^. Neutrophils infiltrating the lungs during *Mp* infection have been shown to contribute to the lung-tissue damage observed in humans, and elevated levels of BAL neutrophils are strongly associated with more severe disease, indicating that the findings in our model are potentially applicable to humans^43–47^.

To our knowledge, this mechanism for VED has not been previously described but should be carefully considered when developing vaccines against bacterial pathogens. This may be particularly important for bacteria that show resistance to killing by neutrophils, such as hypervirulent *Klebsiella pneumoniae*, *Chlamydophila* (formerly *Chlamydia) pneumoniae*, *Streptococcus pyogenes* and some strains of *Staphylococcus aureus*, or against bacteria that show tropism for neutrophils, such as *Anaplasma phagocytophilum*^83–90^. Conversely, vaccine candidates against bacteria that are efficiently cleared by neutrophils (for example *Pseudomonas aeruginosa*, *Listeria monocytogenes*, certain *Salmonella* and *Yersinia* species, *Burkholderia thailandensis*, and certain *Acinetobacter* species) may benefit from the inclusion of lipoproteins in vaccine constructs^91–98^. Indeed, vaccination with recombinant *P. aeruginosa* lipoprotein I (OprI) has shown to be protective against experimental infection with *P. aeruginosa* in mice, and intranasal vaccination with pneumococcal lipoproteins protects against colonization with *Streptococcus pneumoniae*^99,100^. As lipoproteins are found in virtually all bacteria, our findings are broadly relevant, and can serve to inform future vaccine discovery not only for mycoplasmas, but for other bacterial pathogens as well.

The overarching goal of our work was to elucidate the immunological mechanisms underlying *Mp* vaccine-enhanced disease (VED). The elucidation of the immunological mechanisms behind this phenomenon is an important steppingstone in the path towards the development of a safe and efficacious vaccine against *Mp*, as VED has been reported in both humans and animal models and has significantly stymied vaccine development efforts. Here we report for the first time that *Mp* VED is mediated by lipoprotein-induced Th17 recall responses that result in the overexuberant recruitment of neutrophils that exacerbate immunopathology without contributing to the clearance of *Mp* infection. Identifying mechanisms underlying VED is the first step toward overcoming this significant roadblock, and data presented here serve to inform future vaccine development. These experiments illustrate the importance of avoiding the use of native lipoproteins which bear intrinsic adjuvants that trigger Th17-mediated inflammatory events through TLR-2/1 or TLR-2/6 in Mycoplasma vaccines. Using adjuvants that skew memory responses away from Th17, may prove key for the development of a safe and efficacious human *Mp* vaccine. Furthermore, findings here may be generalizable to development of other bacterial vaccines, in that while lipoproteins make good vaccine antigens against bacteria cleared by IL-17A and neutrophils, they instead may be contraindicated when developing vaccines against bacteria resistant to clearance by neutrophils.

## Methods

### Bacterial strains and inoculum culture conditions

*Mycoplasma pneumoniae* strain PI1428 was utilized for all aspects of this study. For infection studies, frozen 50 µL aliquots of low passage *M. pneumoniae* PI1428 were thawed and resuspended in 10 mL of complete Fortified Commercial (FC) medium (20% Heat inactivated horse serum, 5% yeast extract). Cultures were incubated at 37 ˚C with orbital shaking at 120RPM. After 5 h, optical density at 620 nm (OD_620_) was used to estimate colony forming units (CFU) counts per mL of culture. Samples were centrifuged at 5000 x g for 10 min at 4˚C, the supernatant decanted, and the pellet suspended to the desired concentration in fresh FC medium.

### In vivo vaccination/challenge studies

All animal experiments were conducted in accordance with our approved Institutional Animal Care and Use Committee protocol (A20-044). Specific Pathogen Free (SPF) BALB/c mice (8 weeks old) were purchased from Jackson Laboratories (Bar Harbor, ME) and allowed to acclimate for 1 week prior to use. Mice were anesthetized using vaporized isoflurane and intraperitoneally injected with 250 µL of sterile physiological saline (0.9%) for sham vaccination, or 250 µL containing 50 µg of protein from the appropriate bacterial fraction (LAMPs or dLAMPs) as we have previously described^25^. Mice were boosted similarly 21 days after the primary injection. Twenty-one days after the boost, mice were intranasally challenged with 50 µL of FC medium containing 1 × 10^8^ CFU of *M. pneumoniae* PI1428. Remaining challenge inocula were incubated to ensure that appropriate viable titers were administered to all challenged mice. Four days post-infection, mice were humanely sacrificed via cervical dislocation and lungs were immediately harvested for histopathology and mycoplasma recovery. For IL-17A neutralization experiments, mice were vaccinated with LAMPs as described above. Starting 1 day prior to challenge (day −1) and continuing daily until the end of the study period (day 4), mice were intraperitoneally injected with 150 μg/250 μl/dose of either murine monoclonal anti-IL-17A antibody (BioXcell; clone 17F3, *InVivoMAb* anti-mouse IL-17A Cat#. BE0173) or the IgG1 isotype control antibody (BioXCell; clone MOPC-21, *InVivoMAb* IgG1 isotype control, Cat#. BE0083; San Antonio, TX). For the neutrophil depletion studies, mice were vaccinated with LAMPs as described above. Starting 3 days prior to challenge (day-3) and continuing throughout the end of the study period (day 4), mice were intraperitoneally injected daily with 25 μg/250 μl/mouse of the anti-Ly6G antibody (clone 1A8*, InVivoPlus* anti-mouse Ly6G, Cat#. BP0075-1) and with 50 μg/250 μl/mouse of the anti-rat kappa light chain antibody (clone MAR18.5, *InVivoMAb* anti-rat Kappa Ig Light Chain, Cat#. BE0122) every other day to achieve sustained neutrophil depletion as following previously published recommendations^50^. Control animals were treated with 25 μg/250 μl/mouse of the isotype controls (clones 2A3, Cat#. BE0089 and MOPC-21Cat#. BE0083) using the same timeline as 1A8 and MAR18.5 clones. Bronchoalveolar lavages were performed on some animals and used to determine BALF concentrations of IL-17A, TNF-α, IL-1β, IL-6, and KC using commercial murine sandwich ELISA kits (Biolegend, San Diego, CA). Differential cell counts were also performed on BALF collected cells after slide preparation by spinning samples for 5 min at 460 rcf/xg on a Thermo Scientific Cytospin 4 Centrifuge and stained using a Kwik-Diff kit. Five hundred leukocytes were differentially counted per slide (representing one individual animal) to determine leukocyte proportions/ percentages. To determine numbers of specific leukocytes, the number of total leukocytes determined through cell counts on a hemocytometer was multiplied by the proportions received by the differential counts.

### Mycoplasma recovery and histopathology

For mycoplasma recovery, the lower right lobe of the lung was removed and placed into 1 mL of FC medium, vortexed, and incubated for 3 h at 37 °C. The remaining lung tissue was inflated with 10% neutral buffered formalin and allowed to fix for histopathologic evaluation. After 3 h of incubation, *Mycoplasma* recovery samples were passed through a 0.45 µm filter and transferred to new sterile tubes. Quantification of recovery cultures was performed by assessing CCU in 10-fold serial dilutions performed on 96 well tissue culture plates. Samples were incubated for 28 days, and color change was observed and recorded daily. After fixation for 48 h in 10% neutral buffered formalin, tissues were routinely processed into 5um thick sections and stained with H&E. Slides were scored in a blinded fashion by an individual with experience scoring lesions seen in this model, using the following system: 0—no lesions, 1—mild lesions, 2—moderate lesions, 3—marked lesions, 4—severe lesions. Half-step intervals (i.e + 0.5) were used when lesions fell between any two categories. For lungs used for RNAScope in-situ hybridization the lungs were inflated with 1 mL of 10% neutral buffered formalin, allowed to fix at room temperature for 26 h then transferred to 70% ethanol prior to paraffin embedding. RNA in-situ hybridization to label CD4 and IL-17A transcripts was performed according to manufacturer’s instructions utilizing the following probes: RNAscope Mouse probe for il17a in C1 (blue) (#319571) and Mouse CD4-C2 (red) probe (#406841) (ACDBio, Newark, CA).

### Lung digest and flow cytometry sample preparation

Four days after *M. pneumoniae* infection of Sham/LAMPs/dLAMPs-vaccinated mice were humanely euthanized via isoflurane anesthesia followed by cervical dislocation. Mouse lungs were lavaged to reduce the number of neutrophils as we wanted to focus our analysis on lymphocyte populations that are more common in the lung interstitial lesions (perivascular and peribronchiolar). To obtain a single cell suspension from the lung, mouse lungs were collected in 10mLs of DMEM with 20% FBS, 2x L-Glutamine, 1x Non-essential amino acids, 1x glucose, 1X HEPES, 0.5 mg/mL Collagenase type II and 50 units/mL of DNAse I. Lungs were then incubated with gentle agitation at 37 °C for 30 min to allow collagenase digestion, After this initial 30 min incubation, the lungs were then minced into small pieces using scissors and a scalpel blade then returned to incubate in the aforementioned medium for another 20 min to digest. Following the second incubation, the lungs were crushed through a 70um cell strainer into a clean tube using the plunger of a sterile syringe, and cells stuck on the strainer were washed down using additional tissue harvest medium. The single cell suspension was then stored on ice. Cells were pelleted by centrifugation at 500xg for 5 min at 4 °C and resuspended in 5 mL of 1X RBC lysis buffer (Biolegend) until red blood cell lysis was apparent (~2–5 min) then the reaction was stopped by adding 30 mL of PBS and pelleting the cells. The cells were then resuspended in 1 mL PBS and cell counts were determined using a hemocytometer. Cells were then diluted to 1 × 10^7^ cells per mL in PBS. 100 uL of the cell suspension (~1 million cells) were aliquoted to pre-labeled tubes containing samples, single stained controls, unstained controls, and full-minus-one controls. ZombieViolet Dye (Biolegend) was used as a fixable viability marker. Following staining by the ZombieViolet Dye cells were washed then resuspended in FACS buffer and FC receptors were blocked with anti-CD16/32 antibody. The cells were surface stained for CD3 using a BV510 conjugated anti-mouse CD3 antibody (Biolegend, Clone 17 A2, Cat#:100233, dilution 1/200), and CD4 using an APC-Cy7 conjugated anti-mouse CD4 antibody (Biolegend, Clone GK1.5, Cat#: 100413, dilution 1/200). Following surface staining, cells were washed twice with FACS buffer then resuspended in 500uL of FluoroFix Buffer (Biolegend) to fix. Cells were then washed once with FACS and twice with Intracellular Staining Permeabilization Buffer (Biolegend) and stained for intracellular IL-17A with a PE conjugated anti mouse-IL-17A antibody(Biolegend, clone TC11-18H10.1, Cat#: 506904, dilution 1:50). Following the staining incubation, cells were then again washed 2x with intracellular staining buffer, then 2x in FACS buffer. After the final wash, cells were resuspended in 500uL of FACS buffer for analysis using the BD LSRFortessa X-20 Cell Analyzer. Compensation Beads, and single stained controls were used to generate the compensation matrix. Data were analyzed using FlowJo.

### Statistical analyses

Given the nonparametric nature of the lesion score and recovery data, results were analyzed via a nonparametric one-way ANOVA on ranks (Kruskal–Wallis) with a Dunn’s post-hoc test for multiple pairwise comparisons between groups (*α* = 0.05). Analysis of parametric data was done via a parametric one-way ANOVA with a Tukey’s post-hoc test for multiple pairwise comparisons. Analysis of data with only two groups was conducted utilizing a one-tailed Mann–Whitney *U*-test for nonparametric data or a one-tailed *t*-test for parametric data. Linear regression was utilized to establish correlations. Correlation strength is determined based on the correlation coefficient *R*, where *R* = .020 indicates a small effect size and strength of relationship, *R* = 0.40 indicates a moderate effect size and strength of relationship and *R* = 0.60 indicates a large effect size and strength of relationship. All data were analyzed using the GraphPad Prism software, version 8.02 (GraphPad Software, La Jolla California USA).

### Reporting summary

Further information on research design is available in the Nature Research Reporting Summary linked to this article.

## Supplementary information

Mara_Arlind_NPJ_Vax_NPJVACCINES-02008R1_Resubmission_Supplemental Information.pdf

REPORTING SUMMARY

## Data Availability

The data that support the findings of this study are available within the manuscript and supplementary file.
