# Supplementary material for: Vaccination with Mycoplasma pneumoniae membrane lipoproteins induces IL-17A driven neutrophilia that mediates Vaccine-Enhanced Disease
Source: NPJ Vaccines. 2022 Jul 29;7:86. doi: 10.1038/s41541-022-00513-w (PMC9336141; doi:10.1038/s41541-022-00513-w)
Supplement: Supplementary file 1 — Mara_Arlind_NPJ_Vax_NPJVACCINES-02008R1_Resubmission_Supplemental Information.pdf [file 41541_2022_513_MOESM1_ESM.pdf]

Supplementary Information for

Vaccination with *Mycoplasma pneumoniae* membrane lipoproteins induces IL-17A driven neutrophilia that mediates Vaccine-Enhanced Disease

Arlind B. Mara[a,b], Tyler D. Gavitt[a,b], Edan R. Tulman[a,b], Jeremy M. Miller[a,b],Wu He[c], Emily M. Reinhardt[a,d], R. Grace Ozyck[a], Meagan L. Goodridge[a], Lawrence K. Silbart[a,b,e], Steven M. Szczepanek[a,b*] Steven J. Geary[a,b*]

[a] Department of Pathobiology and Veterinary Science, [b] Center of Excellence for Vaccine Research (CEVR), 61 N Eagleville Rd. Unit-3089, Storrs, CT 06269.

[c] Flow Cytometry Facility, Center for Open Research Resources and Equipment, 438 Whitney Rd Ext Unit 1149, Storrs, CT, 06269.

[d] Connecticut Veterinary Medical Diagnostic Laboratory (CVMDL), 61 N Eagleville Rd. Unit-3089, Storrs, CT 06269.

[e] Department of Allied Health, 358 Mansfield Rd, Unit-1101Storrs, CT 06269.

University of Connecticut.

*Corresponding authors

*Steven J. Geary and Steven M. Szczepanek.

Email: steven.geary@uconn.edu, steven.szczepanek@uconn.edu

This PDF file includes:

Figures S1 to S14
Figure Legends
Supplemental References

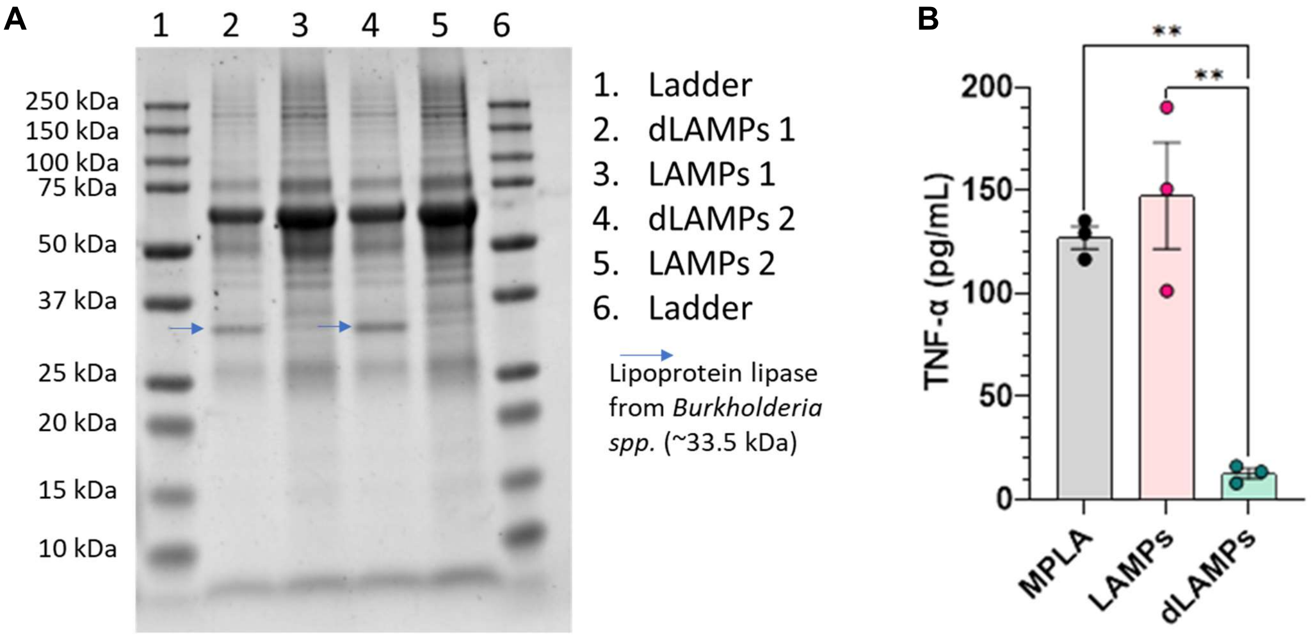

Supplementary Figure 1. (A) Composition of *M. pneumoniae* LAMPs and dLAMPs fractions based on SDS-PAGE analysis and (B) TNF-α expression induced by the LAMPs vs. the dLAMPs fraction in stimulated murine J774A.1 macrophages. Protein profile of the dLAMPs does not differ from that of the LAMPs (with the exception of the additional band at ~33.5kDA representing the added lipoprotein lipase), indicating that lipoprotein lipase treatment does not affect the protein portion of the LAMPs fraction. dLAMPs induce lower expression of TNF-α than LAMPs do in stimulated murine macrophages, indicating that lipoprotein lipase treatment successfully delipidated the *M. pneumoniae* lipoproteins.

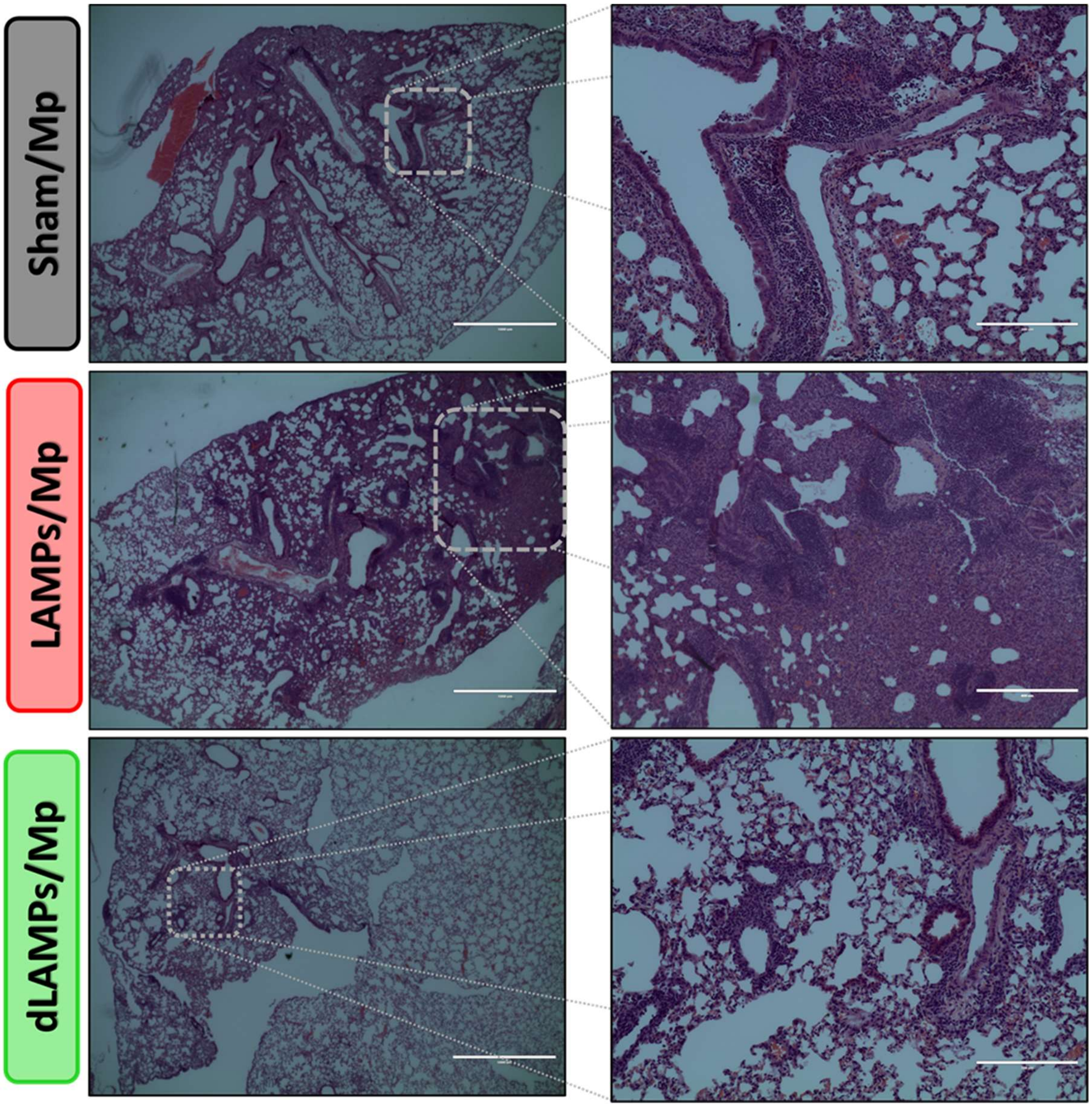

Supplementary Figure 2. ***Vaccination with M. pneumoniae lipoproteins is associated with more severe histopathology upon challenge.*** H&E-stained murine lungs representative of histopathology observed in Sham-vaccinated/Mp-challenged (top), LAMPs-vaccinated/Mp-challenged (middle), and dLAMPs-vaccinated/Mp-challenged (bottom) animals. When compared to Sham- and dLAMPs- vaccinated/Mp-challenged animals, LAMPs-vaccinated/Mp-challenged animals show a more severe suppurative pneumonia as well as larger and denser perivascular and peribronchiolar infiltrates. Perivascular cuffing is more frequently observed in LAMPs-vaccinated/Mp-challenged animals.

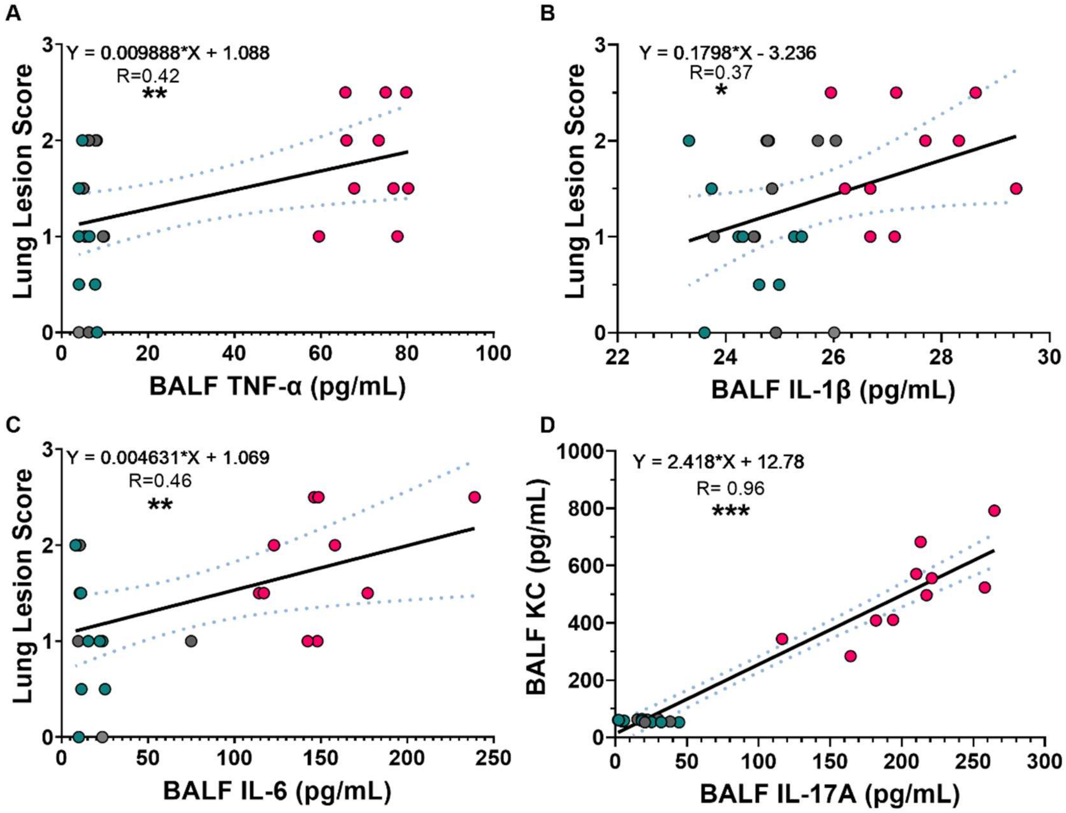

Supplementary Figure 3. Positive correlations between lung lesion scores (disease severity) and bronchoalveolar lavage fluid (BALF) concentrations of (A) TNF-α, (B) IL-1β, (C) IL-6. (D) Positive correlation between BALF IL-17A and BALF KC concentrations. Correlations were determined via linear regression. Correlation strength is determined based on the correlation coefficient R, where R=.20 indicates a small effect size and strength of relationship, R=.40 indicates a moderate effect size and strength of relationship and R=.60 indicates a large effect size and strength of relationship. Dotted lines for linear regression graphs indicate 95% confidence intervals. Each point represents data from an individual animal. * indicates p <0.5, ** indicates p<0.1, *** indicates p<0.01, **** indicates p<0.001.

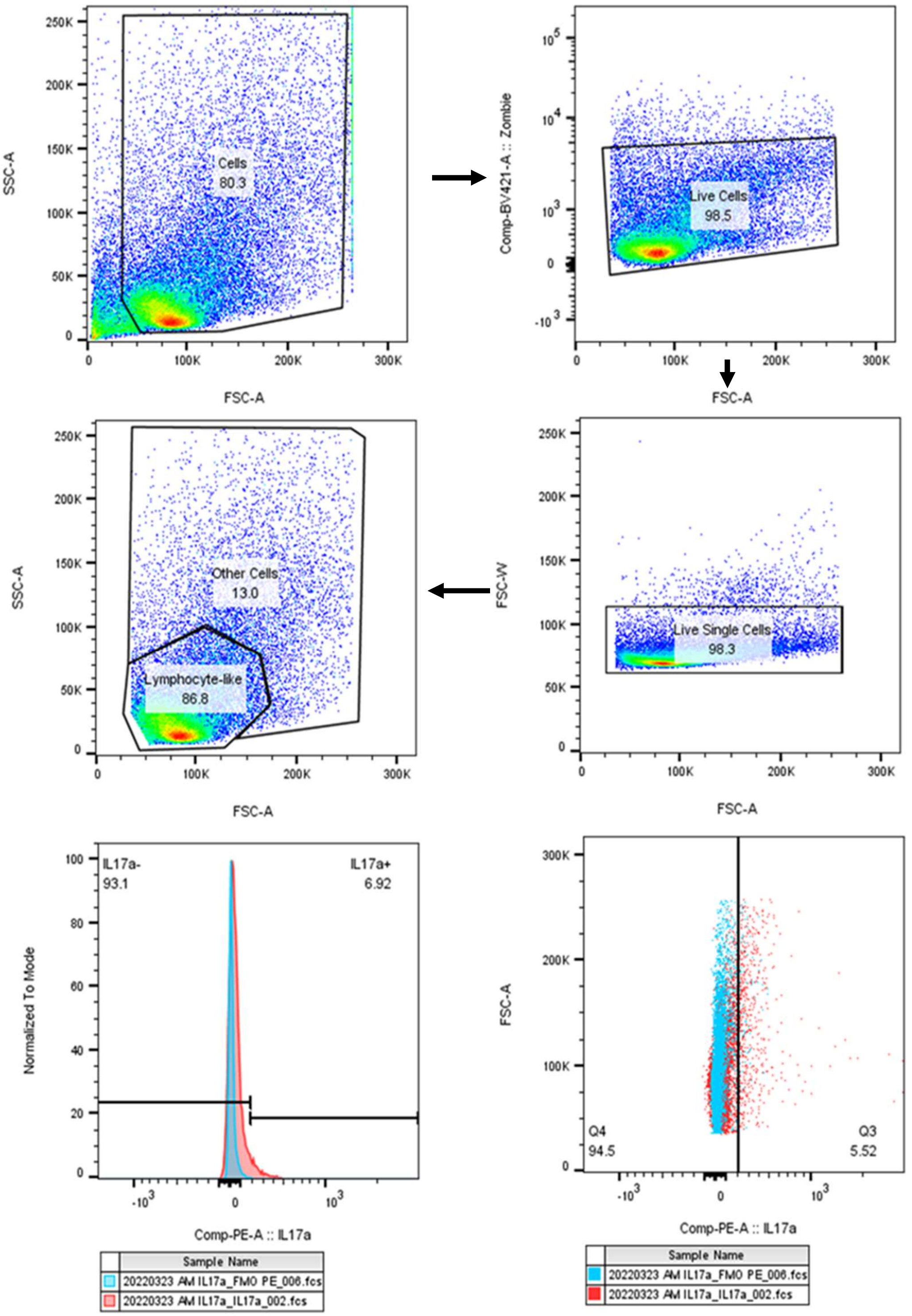

Supplementary Figure 4. Gating Strategy for Flow Cytometry Experiment. Debris was gated out using FSC-A and SSC-A parameters then live cells were identified based on the amount ZombieViolet^{TM} Viability die taken up by the cells. ZombieViolet^{TM} unstained cells used as control for gate placement. Singlets were identified based on FSC-A vs FSC-W, and single cells were then gated as lymphocyte-like or other based on size (FSC-A) and complexity (SSC-A). IL-17A(PE) gate was based on IL-17A/PE Full Minus One-control.

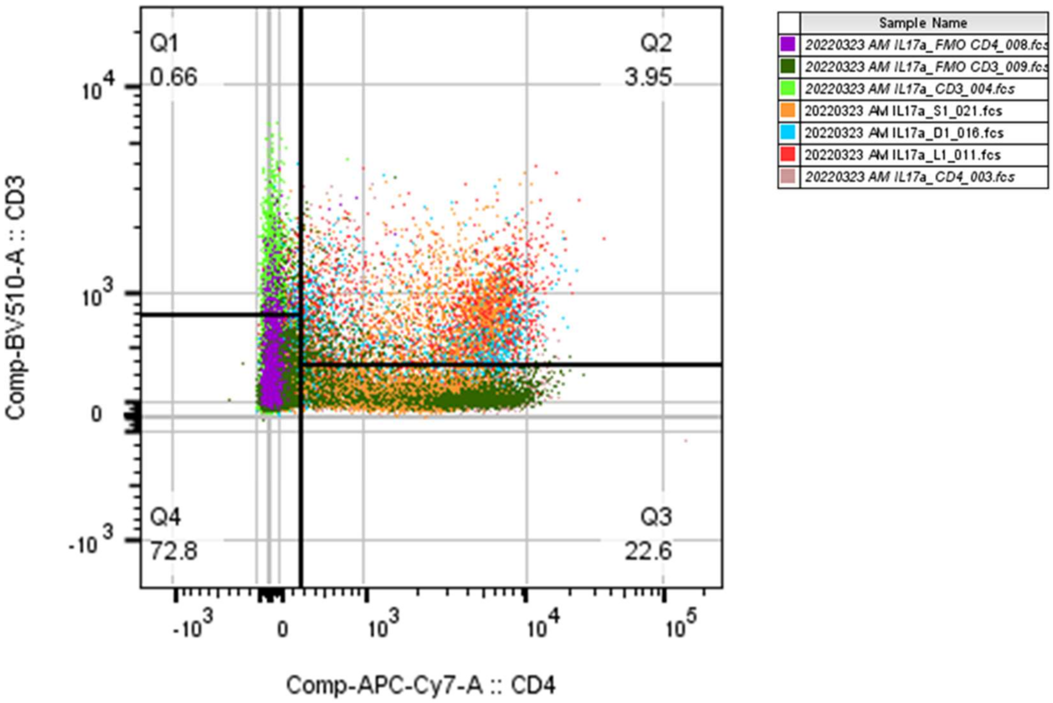

Supplementary Figure 5. Gating Strategy for Lymphocyte-like subsets. Gates for CD3 and CD4 were based on Full Minus One (FMO) and Single Stain controls for CD3 and CD4.

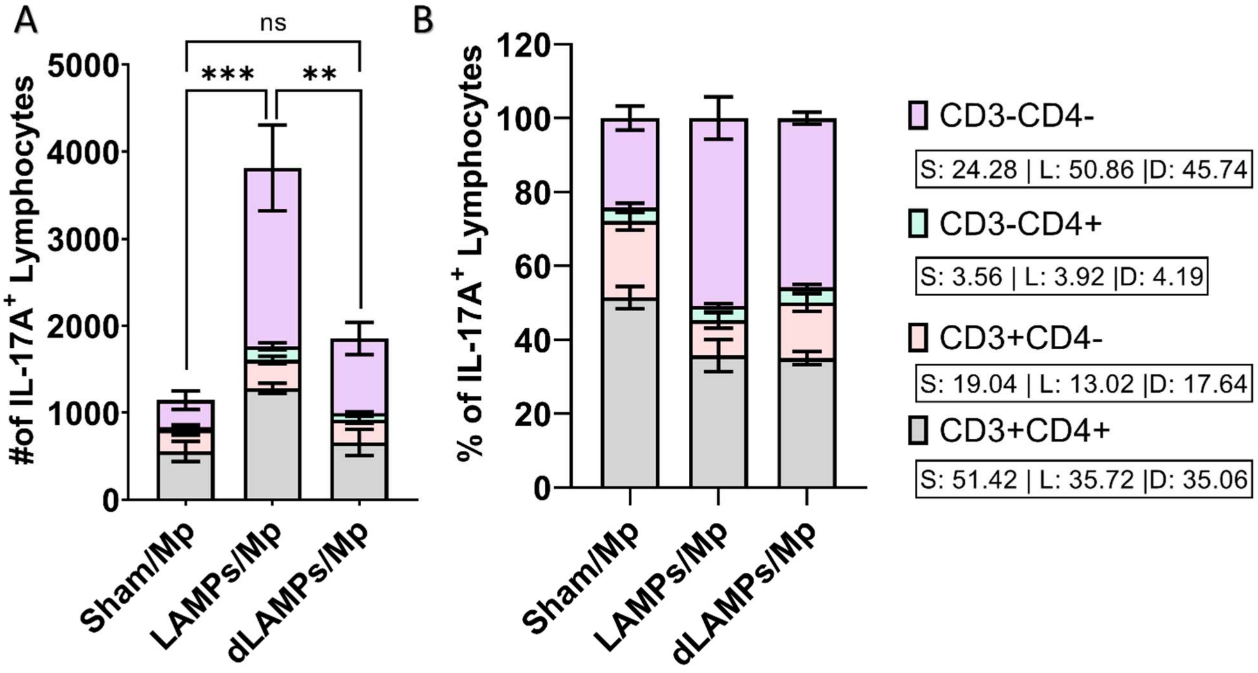

Supplementary Figure 6. Numbers (A) and percentages (B) of IL-17A positive lymphocyte-like $CD3^{+/-}CD4^{+/-}$ subsets. * indicates p <0.5, ** indicates p<0.1, *** indicates p<0.01, ****. Error bars indicate mean and SEM.

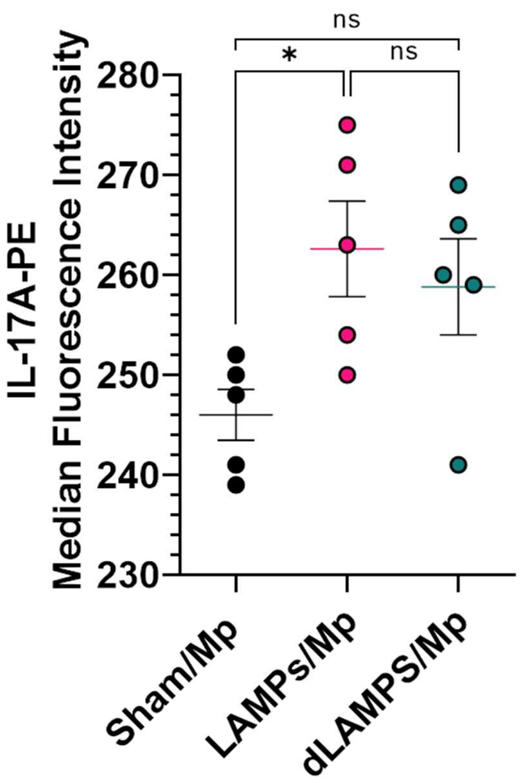

Supplementary Figure 7. IL-17A:PE Median Fluorescence Intensities in IL-17A+ live-single-cells from the lungs of Sham-, LAMPs-, and dLAMPs-vaccinated/Mp-challenged animals. * indicates p <0.5, ** indicates p<0.1, *** indicates p<0.01, ****. Error bars indicate mean and SEM. Each point represents data from an individual animal.

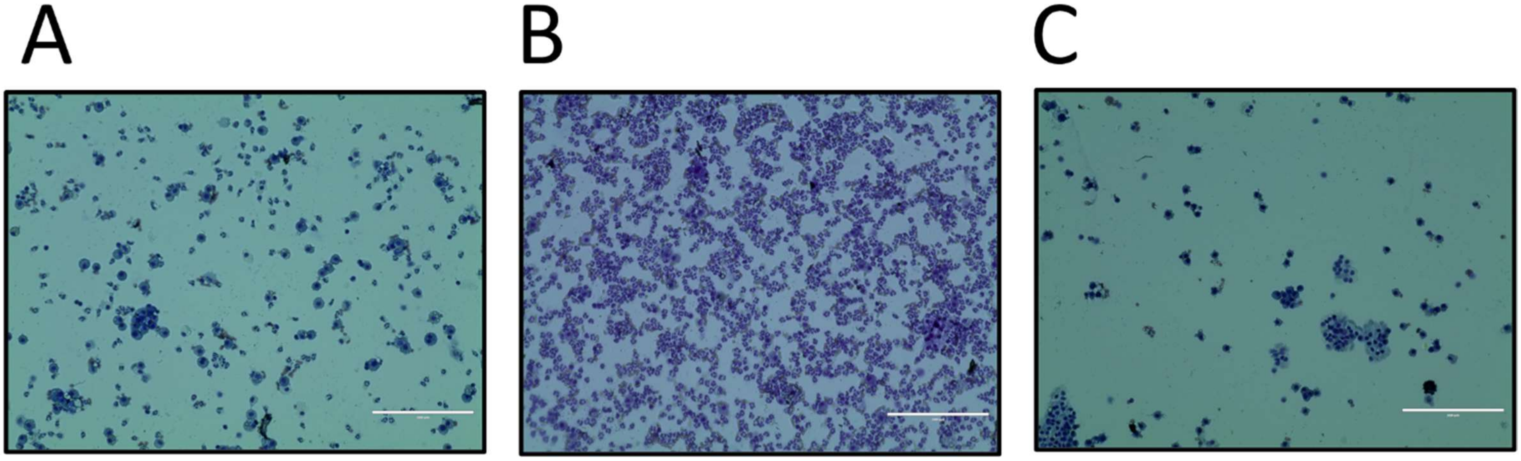

Supplementary Figure 8. H&E stained bronchoalveolar lavage fluid cells from (A) Sham-vaccinated/Mp-challenged animal, (B) LAMPs-vaccinated/Mp-challenged animal, and (C) dLAMPs-vaccinated animal.

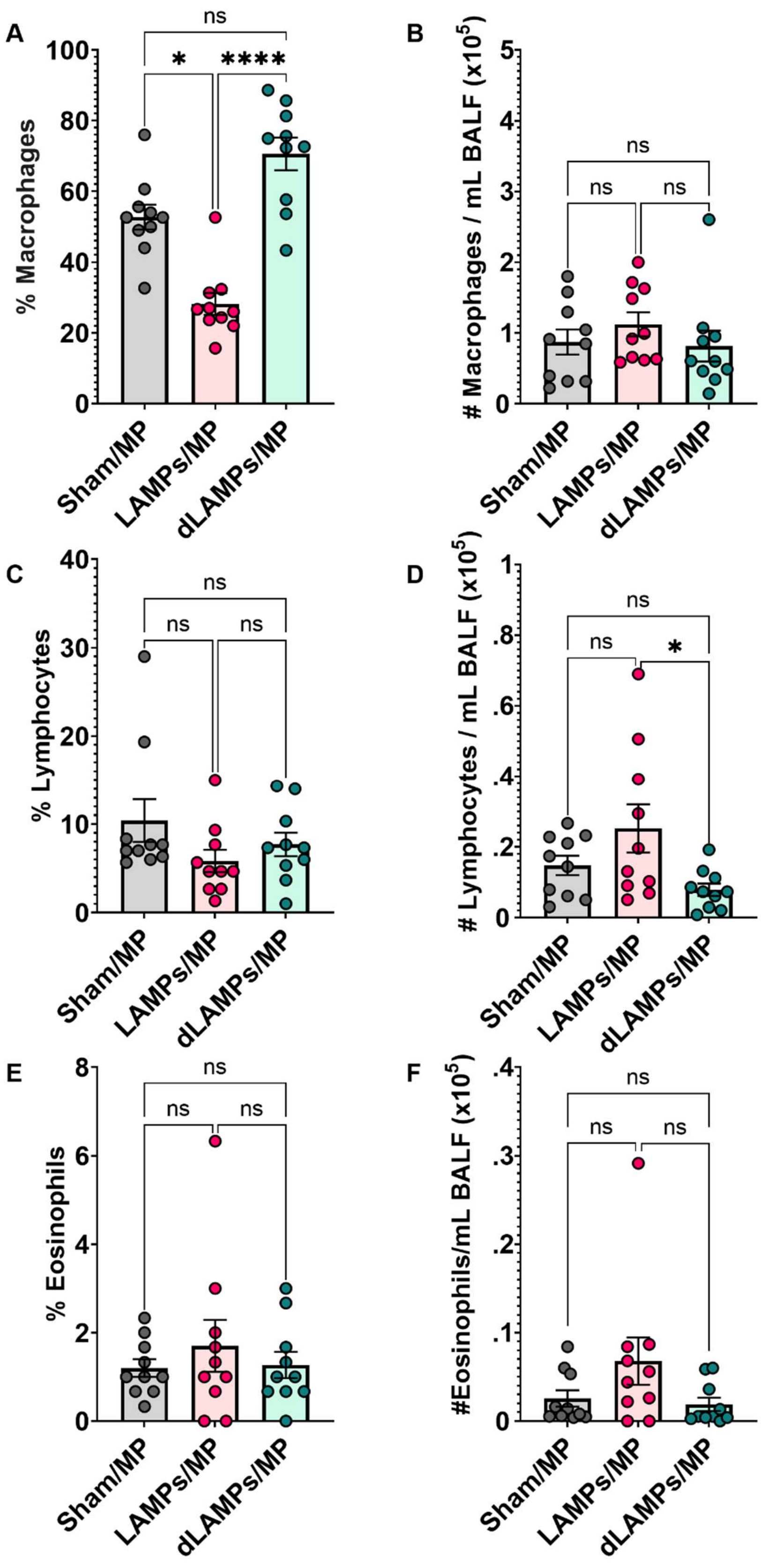

Supplementary Figure 9. Bronchoalveolar fluid macrophage proportions of (A) macrophages, (C) lymphocytes, and (E) eosinophils and calculated numbers of (B) macrophages (D) lymphocytes and (F) eosinophils in vaccinated-then-challenged animals. Non-parametric proportion data were analyzed via a Kruskal-Wallis one-way ANOVA on ranks with a Dunn's *post-hoc* test for multiple comparisons, while parametric cell num bers were analyzed via a one-way ANOVA with a Tukey's *post-hoc.* * indicates p <0.5, ** indicates p<0.1, *** indicates p<0.01, **** indicates p<0.001. Each point represents data from an individual animal.

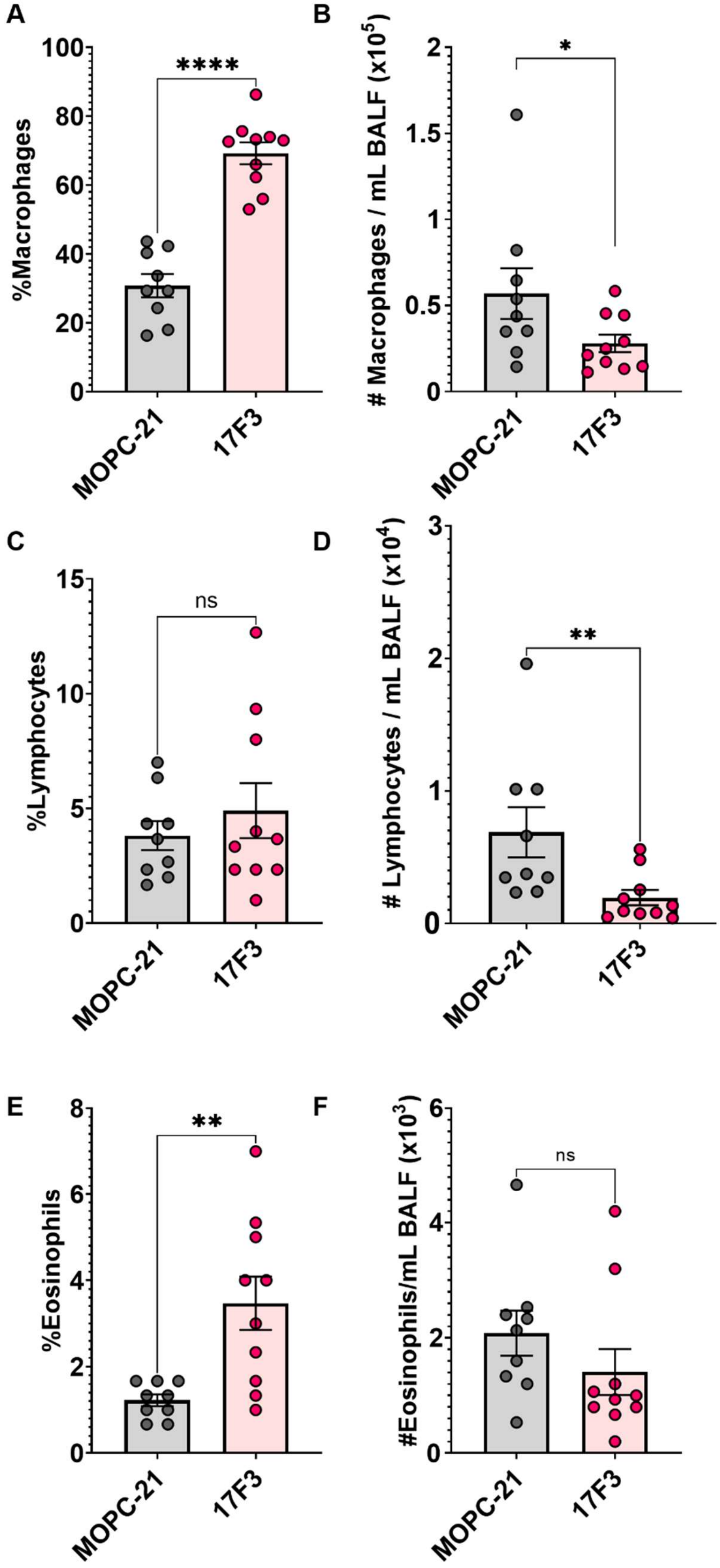

Supplementary Figure 10. Bronchoalveolar fluid macrophage proportions of (A) macrophages, (C) lymphocytes, and (E) eosinophils and calculated numbers of (B) macrophages (D) lymphocytes and (F) eosinophils in LAMPs-vaccinated/Mp-challenged animals receiving either an anti-IL-17A antibody (17F3) or isotype control (MOPC-21). Non-parametric proportion data were analyzed via non-parametric Mann-Whiteny U tests, while parametric cell numbers were analyzed via parametric unpaired t-tests.* indicates p <0.5, ** indicates p<0.1, *** indicates p<0.01, **** indicates p<0.001. Each point represents data from an individual animal.

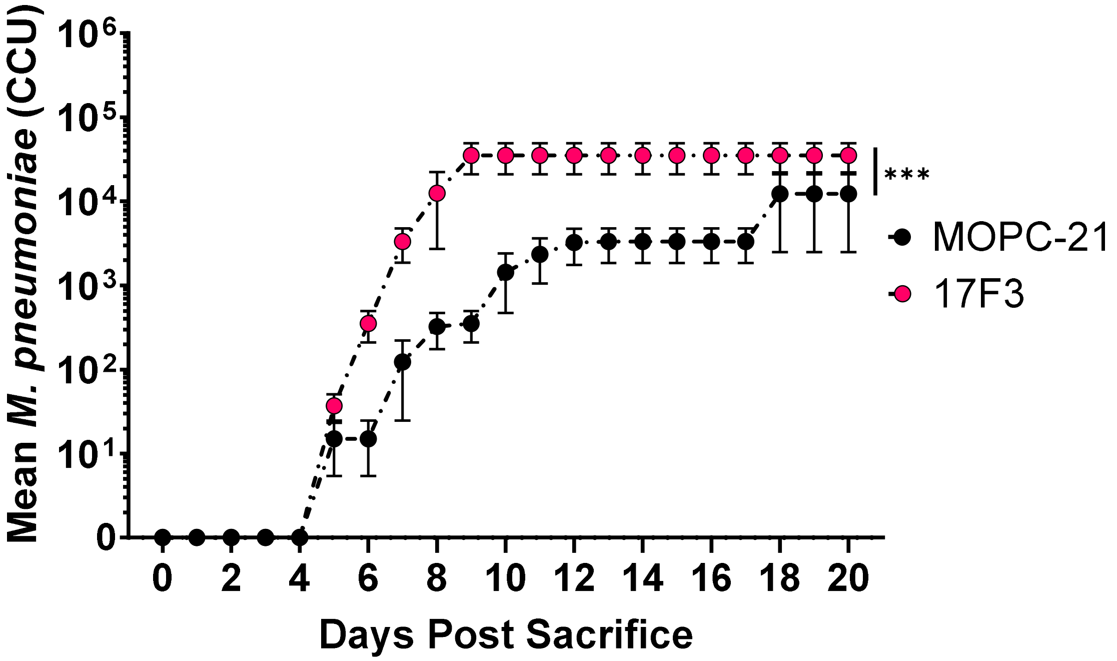

Supplementary Figure 11. Mean *M. pneumoniae* titers in CCU displayed as a time course of growth kinetics after recovery from LAMPs-vaccinated/Mp-challenged animals treated with an anti-IL-17A antibody or isotype control. Given CCUs are assessed via 10-fold dilutions, the dynamics of growth to an endpoint dilution can also be indicative of relative Mp titers recovered. A two-way ANOVA was conducted to assess if there is a difference between time dependent Mp growth kinetics between the two differentially treated groups. Point represent the mean CCU of the group while error bars indicate the SEM. * indicates p <0.5, ** indicates p<0.1, *** indicates p<0.01, **** indicates p<0.001.

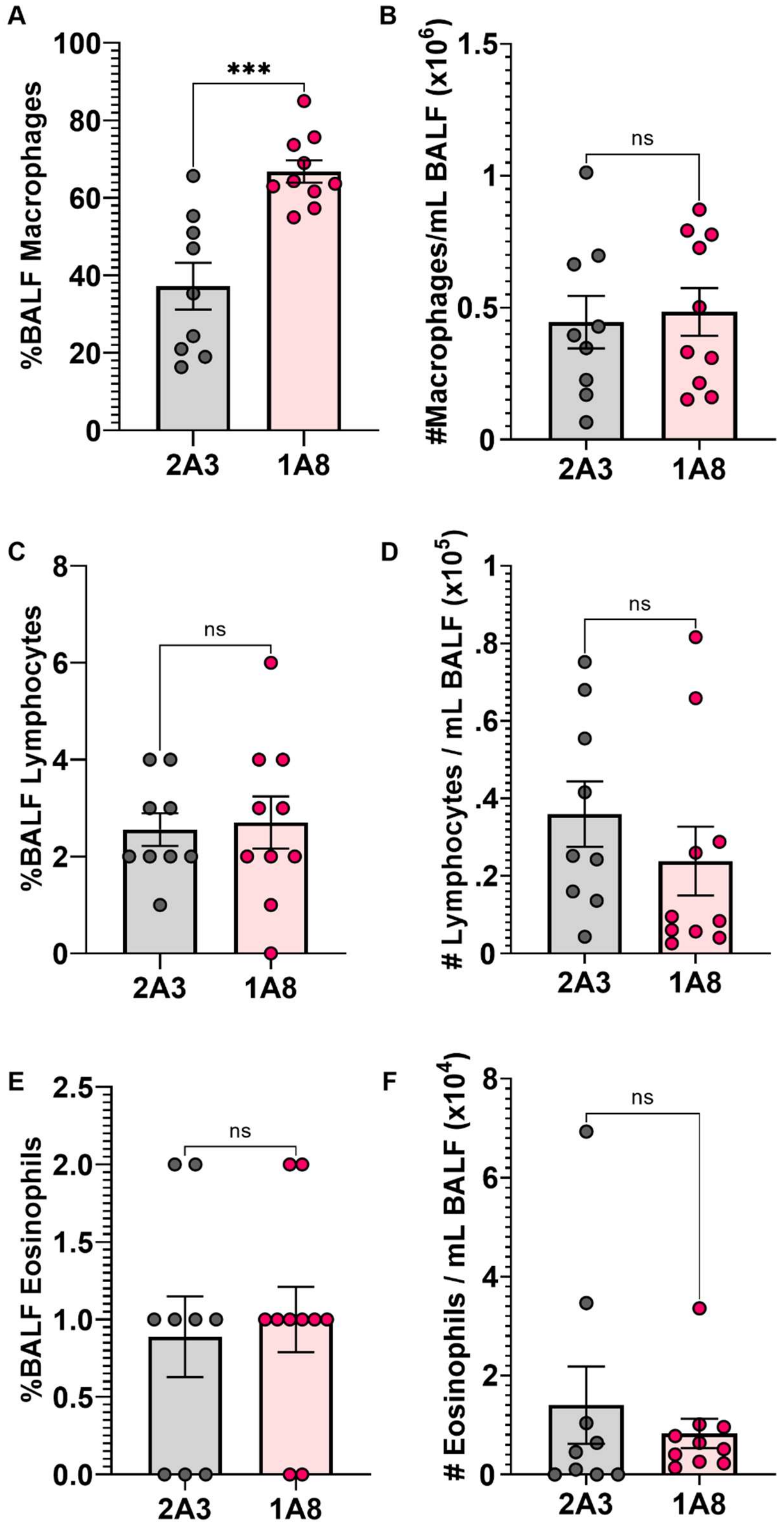

Supplementary Figure 12. Bronchoalveolar fluid macrophage proportions of (A) macrophages, (C) lymphocytes, and (E) eosinophils and calculated numbers of (B) macrophages (D) lymphocytes and (F) eosinophils in LAMPs-vaccinated/Mp-challenged animals receiving either neutrophil depleting antibodies (1A8) or isotype controls (2A3). Non-parametric proportion data were analyzed via non-parametric Mann-Whitney U tests, while parametric cell numbers were analyzed via parametric unpaired t-tests.* indicates p <0.5, ** indicates p<0.1, *** indicates p<0.01, **** indicates p<0.001. Each point represents data from an individual animal.

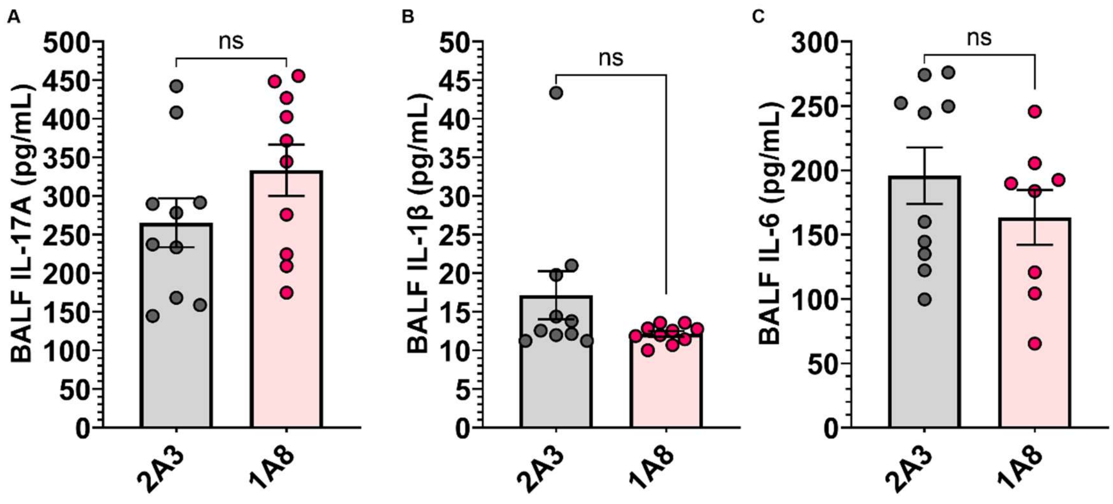

Supplementary Figure 13. Bronchoalveolar lavage fluid concentrations of (A) IL-17A, (B) IL-1β, (C) IL-6 of LAMPs-vaccinated/Mp-challenged animals that received either neutrophil depleting antibodies (1A8) or isotype controls (2A3). Data were analyzed via parametric unpaired t-tests. .* indicates p <0.5, ** indicates p<0.1, *** indicates p<0.01, **** indicates p<0.001. Each point represents data from an individual animal.

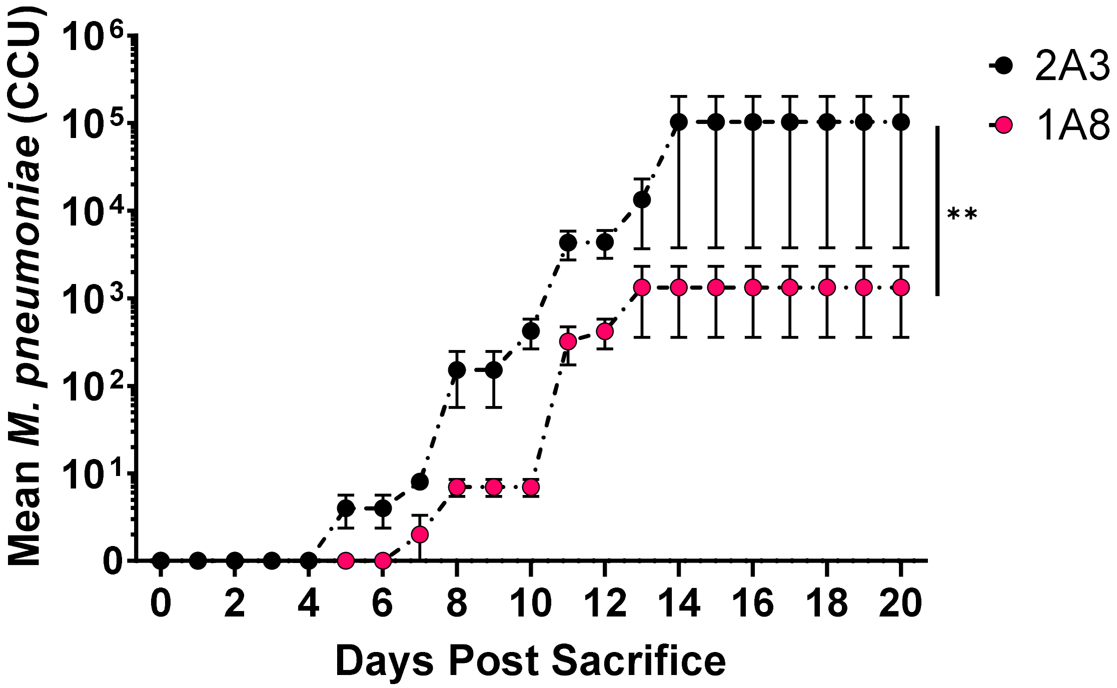

Supplementary Figure 14. Mean *M. pneumoniae* titers in CCU displayed as a time course of growth kinetics after recovery from LAMPs-vaccinated/Mp-challenged animals treated with neutrophil depleting antibodies (1A8) or isotype controls (2A3). Given CCUs are assessed via 10-fold dilutions, the dynamics of growth to an endpoint dilution can also be indicative of relative Mp titers recovered. A two-way ANOVA was conducted to assess if there is a difference between time dependent Mp growth kinetics between the two differentially treated groups. Point represent the mean CCU of the group while error bars indicate the SEM. * indicates $p < 0.5$, ** indicates $p < 0.1$, *** indicates $p < 0.01$, **** indicates $p < 0.001$.

Supplemental References

1. Yingxia Zheng, Lei Sun, Ting Jiang, Dongqing Zhang, Dongyi He, Hong Nie, "TNF*α* Promotes Th17 Cell Differentiation through IL-6 and IL-1*β* Produced by Monocytes in Rheumatoid Arthritis", *Journal of Immunology Research*, vol. 2014. https://doi.org/10.1155/2014/385352
2. Hwang YS, Jeong M, Park JS, Kim MH, Lee DB, Shin BA, Mukaida N, Ellis LM, Kim HR, Ahn BW, Jung YD. Interleukin-1beta stimulates IL-8 expression through MAP kinase and ROS signaling in human gastric carcinoma cells. Oncogene. 2004 Aug 26;23(39):6603-11. doi: 10.1038/sj.onc.1207867. PMID: 15208668.
3. Osawa Y, Nagaki M, Banno Y, Brenner DA, Asano T, Nozawa Y, Moriwaki H, Nakashima S. Tumor necrosis factor alpha-induced interleukin-8 production via NF-kappaB and phosphatidylinositol 3-kinase/Akt pathways inhibits cell apoptosis in human hepatocytes. Infect Immun. 2002 Nov;70(11):6294-301. doi: 10.1128/IAI.70.11.6294-6301.2002. PMID: 12379708; PMCID: PMC130316.
